# Global burden of lip and oral cavity cancer across adults aged ≥45 years from 1990 to 2021 and projections to 2050

**DOI:** 10.18332/tid/211972

**Published:** 2025-12-10

**Authors:** Minsi Li, Cen Wang, Yi Wei, Xiaofeng Qin, Wenhua Huang, Bo Zhou, Xuanping Huang

**Affiliations:** 1 College of Stomatology, Guangxi Medical University, Nanning, China; 2 Guangxi Key Laboratory of Oral and Maxillofacial Rehabilitation and Reconstruction, Nanning, China; 3 Guangxi Clinical Research Center for Craniofacial Deformity, Guangxi Medical University, Nanning, China; 4 School of Information and Management, Guangxi Medical University, Nanning, China; 5 Department of Radiation Oncology, The First Affiliated Hospital of Guangxi Medical University, Nanning, China; 6 School of Continuing Education, Guangxi Medical University, Nanning, China

**Keywords:** DALY, risk factors, global burden of disease, sociodemographic index, lip and oral cavity cancer

## Abstract

**INTRODUCTION:**

As global population aging intensifies, the burden of lip and oral cavity cancer (LOCC) among middle-aged and older adults continues to worsen. This research systematically analyzed global LOCC burden trends among adults aged ≥45 years, aiming to inform evidence-based policy and public health strategies.

**METHODS:**

Key metrics were obtained from the GBD 2021 database including age-standardized incidence rate (ASIR), age-standardized prevalence rate (ASPR), age-standardized mortality rate (ASMR) and age-standardized DALY rate (ASDR). Their correlations with the sociodemographic index (SDI) were explored. Joinpoint models assessed trends via annual percent change (APC) and average annual percent change (AAPC). Bayesian Age-Period-Cohort (BAPC) models projected future trends.

**RESULTS:**

From 1990 to 2021, globally, the ASPR (EAPC=0.77; 95% CI: 0.72–0.82) and ASIR (EAPC=0.35; 95% CI: 0.29–0.41) of LOCC among adults aged ≥45 rose significantly, while ASMR (EAPC= -0.15; 95% CI: -0.20 – -0.09) and ASDR (EAPC= -0.25; 95% CI: -0.31 – -0.20) rates declined. Regionally, all SDI quintiles experienced rising ASPR and ASIR, with middle SDI regions showing the fastest growth. Low-middle and low SDI areas saw increases in ASMR and ASDR. A notable correlation was identified between ASPR, ASIR and SDI. East Asia and Oceania had the most severe increases in ASPR/ASIR and ASMR/ASDR, respectively. Males bore a greater burden than females. Population growth and epidemiological shifts drove the rise in ASIR and ASPR, with alcohol and tobacco use as key mortality contributors. Projections estimate ASPR will reach 61.81 (95% UI: 37.31–86.30) and ASIR 17.09 (95% UI: 11.95–22.23) by 2050, with ASMR and ASDR expected to initially decline before rising again.

**CONCLUSIONS:**

The study highlights the need for early prevention, especially in high-risk regions and among male adults aged ≥45 years, and emphasizes the importance of considering multiple factors in public health interventions for effective disease management.

## INTRODUCTION

Lip and oral cavity cancer (LOCC), a major type of head and neck malignant tumor, primarily manifests as squamous cell carcinoma, posing a severe threat to patients’ quality of life and prognosis^1^. According to 2022 global cancer statistics from the International Association of Cancer Registries (IACR), oral cancer ranks 16th in incidence and 15th in mortality among 36 common malignancies, with an annual total of 389485 newly diagnosed cases and 188230 deaths reported^2^. Notably, in countries with lower human development indices, the ASIR of oral cancer in males ranks third, following prostate and lung cancers^2^.

Tobacco use and alcohol consumption remain the two primary behavioral risk factors for the onset and progression of oral cancer^3^. Tobacco is consumed in various forms, including smoking, snuff, and chewing tobacco^4^. Since 1985, the International Agency for Research on Cancer has classified all forms of tobacco as carcinogenic to humans^5^, with sufficient evidence supporting their oral cavity-specific carcinogenicity^6^. Among these, chewing tobacco – particularly when combined with betel quid – poses a significant risk, as the nitrosamines in betel nuts interact with saliva to form carcinogenic compounds, often serving as the primary etiological factor for oral lesions^7^. In contrast, smoking drives malignant transformation through exposure to carcinogenic compounds in tobacco smoke, which trigger genetic mutations and disturb cellular homeostasis^8^.

Age is a significant factor influencing LOCC incidence. Findings from the GBD study underscore that individuals aged 45–74 years exhibit the highest risk of mortality from oral cancer, with this age group constituting the predominant proportion of oral cancer-related deaths^9^. Chinese epidemiological data indicate a peak incidence at 65–69 years^10^, whereas a cohort study in Tehran, Iran, revealed that 89% of patients were aged >40 years, with an average age at diagnosis of 58.8 years^11^. These findings collectively suggest that middle-aged and elderly populations are key targets for LOCC prevention and control. According to WHO forecasts, the world’s population aged ≥60 years will surge from 12% in 2015 to 22% by 2050^12^. The aging process, accompanied by immune function decline and multimorbidity, may further exacerbate the burden of LOCC. However, prospective studies on the long-term epidemiological trends and future disease burden of LOCC in the key demographic group aged ≥45 years remain scarce.

The GBD study, as a globally authoritative health assessment project, provides scientific evidence for disease prevention and policy formulation by integrating multisource health data^13^. Utilizing GBD 2021, our research conducts a comprehensive longitudinal analysis of LOCC burden trends among adults aged ≥45 years worldwide from 1990 to 2021, with projections extending to 2050. Our study addresses critical gaps in epidemiological research on LOCC in this age group, offering evidence-based guidance for tailored public health interventions.

## METHODS

### Data sources

All data in this research were obtained from the GBD 2021, which compiles disease burden data spanning 371 diseases and 88 risk factors across 204 countries and regions worldwide, and employs a unified methodology for data collection and indicator assessment^13^. The case definition for LOCC in the GBD study is based on the International Classification of Diseases,10th Revision (ICD-10): C00-C08.9, D10.0-D10.5, D11-D11.9, and 9th edition (ICD-9): 140–145.9; 210.0–210.6; 235.0^14^. Crucially, the GBD modeling process generates 1000 posterior draws for each metric. The final estimates provided in the database represent the mean of these draws, and the 95% uncertainty intervals (UIs) are defined by the 2.5th and 97.5th percentiles. It should be noted that the GBD estimation strategy comprehensively addresses missing and non-representative raw data through its proprietary modeling algorithms (e.g. spatiotemporal smoothing and cause of death ensemble modeling). As such, the dataset we analyzed provides complete estimates for all locations, years, and demographic groups without requiring further imputation on our part. This research focused on extracting LOCC data for adults aged ≥45 years from 1990–2021 to evaluate their epidemiological trends.

To clarify the reporting of uncertainty in this study, we adhere to the following convention: Uncertainty intervals (UIs) are reported for all estimates directly obtained from the GBD database (e.g. ASRs), representing the comprehensive uncertainty from the GBD modeling pipeline. In contrast, confidence intervals (CIs) are reported for metrics derived from our subsequent statistical analyses (e.g. EAPC, APC, AAPC), representing the uncertainty inherent to those specific models.

The extracted data encompassed a diverse range of dimensions. Firstly, in terms of demographic characteristics, it covered age groups (specifically adults aged ≥45 years), gender, and a time span from 1990 to 2021. Regarding regional classification, the data encompassed 204 countries and territories globally, which included WHO member states, five SDI regions, and 21 GBD regions.

### Analysis indicators

To remove the confounding effect of age structure disparities on the results, age-standardized processing was applied to the disease burden data. The ASPR, ASIR, ASMR and ASDR (with their 95% UIs) were computed using the age structure of the GBD world standard population as the reference. These indicators were employed to compare the magnitude of disease burden among diverse regions and populations.

### Statistical methods

The disease burden data were analyzed via R(v4.3.3). Following data cleaning and organization, we conducted statistical analyses and generated data visualizations by employing R packages including *dplyr*, *officer*, and *ggplot2*. Significance in statistics was defined by p<0.05.

*Age group analysis*

Based on the age-categorization criteria of the GBD 2021, the research population was divided into 11 age groups: 45–49, 50–54, 55–59, 60–64, 65–69, 70–74, 75–79, 80–84, 85–89, 90–94, and ≥95 years. Changes in LOCC disease burden dynamics among different age strata and sexes were explored.

*Trend analysis*

We used R software to calculate and visually display the annual percentage change in disease burden (LOCC) from 1990 to 2021, stratified by age, sex, region, and country. The estimated annual percentage change (EAPC) was derived from a log-linear regression model [ln(Y) = α + βX + ε], using the formula EAPC = 100 × [exp(β) - 1], where β is the regression coefficient, X represents the year, and Y is the natural logarithm of the age-standardized rate. The 95% confidence interval (CI) of the EAPC was used to evaluate statistical significance: an EAPC and its lower 95% CI >0 indicated an increasing trend, whereas values ≤0 indicated a downward trend.

To assess the robustness of the EAPC, we compared its values with the average annual percentage change (AAPC) obtained from the Joinpoint regression model. The AAPC provides a summary trend over a fixed interval by combining the segment-specific trends from the Joinpoint model. A high level of agreement between the EAPC (a single linear assumption) and the AAPC (a model-allowed nonlinear trend) indicates that the estimated long-term trend is stable and not overly sensitive to the choice of model. The joinpoint regression model was utilized to examine temporal trends from 1990 to 2021, identifying inflection points via Monte Carlo permutation tests. The AAPC and APC for each time segment were calculated to characterize overall trends, capture nonlinear patterns in disease burden, and pinpoint specific inflection points. When the 95% CI encompassed zero, the trend was considered to be stable; AAPC or APC significantly >0 or <0 indicated upward or downward trends, respectively. Model fitting and the calculations for AAPC/APC were conducted utilizing Joinpoint software, with subsequent visualizations generated in R.

*Age-period-cohort analysis*

The research utilized an age-period-cohort model to analyze drivers of disease burden changes, distinguishing age effects (lifespan-related risk variations), period effects (short-term impacts from external factors like medical advances or policies), and cohort effects (long-term disparities due to birth cohort-specific risk exposures). To address the model’s identifiability issue (age + period = cohort), we applied the intrinsic estimator (IE) constraint, which provides a unique and stable solution through principal component decomposition. Given the count nature and potential overdispersion of the outcome data (e.g. incident cases), we employed a negative binomial regression framework for the age-period-cohort model. Implemented in R (v4.3.3) using orthogonal decomposition to isolate linear and nonlinear components, parameters were estimated via weighted least squares, and model fit was evaluated using the Wald χ^2^ test.

*Decomposition analysis*

The alterations in disease burden were partitioned into three primary components: age, population and epidemiological transitions. The proportional contributions of various factors were analyzed to identify key drivers of disease burden shifts.

*Correlation analysis*

Pearson correlation analysis was utilized to evaluate the relationships between the SDI and age-standardized rates of LOCC burden. To account for multiple testing, the false discovery rate (FDR) was controlled using the Benjamini-Hochberg procedure, with a significance threshold set at p<0.05. Additionally, the share of LOCCs linked to five key risk factors (behavioral risk, tobacco use, alcohol use, tobacco chewing, and smoking) was analyzed.

### Predictive analysis

The BAPC model was used to estimate trends in LOCC among adults aged ≥45 years from 2022 to 2050. This model’s strength is that it employs second-order random walk priors to even out the effects of age, period and cohort, mitigating overfitting. Specifically, we specified second-order random walk (RW2) priors for the age, period, and cohort parameters to enforce smoothness across these dimensions. This approach regularizes the estimates by penalizing large deviations between adjacent groups, which is particularly advantageous for forecasting. The projections from the BAPC model are presented with their 95% UIs, which propagate the uncertainty from the input GBD 2021 estimates into the future. Model robustness was validated via cross-validation to ensure reliable predictions.

## RESULTS

### Global burden

In 2021, the ASIR of LOCC in individuals aged ≥45 years was 15.64 (95% UI: 14.20–16.83) per 100000 people, the ASPR was 53.65 (95% UI: 49.31–57.49) per 100000 people, the ASMR was 8.06 (95% UI: 7.22–8.74) per 100000 people, and the ASDR was 203.60 (95% UI: 183.29–220.79) per 100000 person-years. This implies that in 2021, there were 372367.03 (95% UI: 338820.50–400693.20) new LOCC cases, 1291322.89 (95% UI: 1188272.15–1383417.94) existing LOCC patients, 190500.56 (95% UI: 170989.68–206482.75) LOCC-related deaths, and 4898440.91 (95% UI: 4413928.41–5311560.59) disability-adjusted life years (DALYs) attributed to LOCC. From 1990 to 2021, the ASMR (EAPC= -0.15; 95% CI: -0.20 – -0.09) and ASDR (EAPC= -0.25; 95% CI: -0.31 – -0.20) of the LOCC significantly decreased, whereas the ASIR (EAPC=0.35; 95% CI: 0.29–0.41) and ASPR (EAPC=0.77; 95% CI: 0.72–0.82) significantly increased (Table 1).

**Table 1:** Age-standardized LOCC burden results for the global population, five SDI regions, and 21 GBD regions

*Location*	*ASPR*			*ASIR*			*ASMR*			*ASDR*		
	*1990 (95% UI)*	*2021 (95% UI)*	*EAPCs (95% CI)*	*1990 (95% UI)*	*2021 (95% UI)*	*EAPCs (95% CI)*	*1990 (95% UI)*	*2021 (95% UI)*	*EAPCs (95% CI)*	*1990 (95% UI)*	*2021 (95% UI)*	*EAPCs (95% CI)*
**Global**	42.85 (40.81–44.78)	53.65 (49.31–57.49)	0.77 (0.72–0.82)	13.91 (13.12–14.65)	15.64 (14.20–16.83)	0.35 (0.29–0.41)	8.24 (7.70–8.77)	8.06 (7.22–8.74)	-0.15 (-0.20– -0.09)	213.82 (201.21–227.41)	203.60 (183.29–220.79)	-0.25 (-0.31– -0.20)
**SDI**												
High	78.78 (74.19–83.08)	86.21 (79.97–91.42)	0.44 (0.36–0.51)	17.77 (16.67–18.76)	17.79 (16.30–18.97)	0.08 (0.03–0.14)	6.62 (6.20–6.95)	5.27 (4.78–5.61)	-0.71 (-0.77– -0.65)	173.52 (164.13–182.50)	129.74 (120.60–137.27)	-0.92 (-0.96– -0.88)
High-middle	35.66 (33.24–38.22)	46.69 (41.63–51.91)	0.89 (0.82–0.96)	11.88 (11.16–12.58)	12.57 (11.24–13.87)	0.10 (0.02–0.19)	6.72 (6.32–7.09)	5.42 (4.84–5.97)	-0.84 (-0.91– -0.77)	176.82 (167.30–186.25)	136.94 (123.45–150.90)	-1.02 (-1.10– -0.94)
Middle	23.06 (21.26–24.93)	41.00 (36.20–46.10)	1.87 (1.73–2.01)	10.14 (9.32–11.00)	13.31 (11.71–14.92)	0.84 (0.74–0.94)	7.64 (6.99–8.30)	7.66 (6.75–8.54)	-0.07 (-0.12– -0.02)	188.88 (173.60–205.19)	187.54 (165.72–209.26)	-0.11 (-0.16– -0.05)
Low-middle	33.81 (29.60–38.48)	50.27 (43.63–56.96)	1.24 (1.09–1.38)	17.39 (15.11–19.91)	21.35 (18.41–24.27)	0.59 (0.50–0.68)	14.30 (12.39–16.43)	15.50 (13.34–17.62)	0.19 (0.13–0.25)	365.02 (318.44–418.99)	387.28 (332.45–441.62)	0.13 (0.08–0.19)
Low	23.57 (19.82–27.52)	31.61 (26.18–37.50)	0.79 (0.63–0.95)	12.75 (10.73–14.83)	14.85 (12.35–17.50)	0.33 (0.22–0.44)	10.71 (9.07–12.48)	11.53 (9.64–13.56)	0.09 (0.01–0.18)	271.74 (230.34–316.41)	281.06 (234.05–331.41)	-0.06 (-0.14–0.03)
**GBD regions**												
East Asia	13.41 (11.52–15.40)	34.11 (27.55–41.72)	3.40 (3.24–3.56)	5.79 (4.93–6.68)	9.38 (7.52–11.54)	1.84 (1.66–2.03)	4.24 (3.60–4.90)	4.21 (3.37–5.18)	0.10 (-0.03–0.23)	102.61 (86.89–119.46)	100.01 (79.68–123.89)	0.05 (-0.09–0.18)
Southeast Asia	27.95 (23.74–32.48)	42.72 (35.04–51.64)	1.27 (1.09–1.45)	11.62 (9.93–13.48)	13.82 (11.44–16.48)	0.49 (0.36–0.62)	8.67 (7.40–10.06)	8.58 (7.13–10.11)	0.00 (-0.09–0.10)	201.18 (171.65–233.98)	194.60 (162.53–229.57)	-0.03 (-0.11–0.05)
Oceania	12.99 (9.38–17.13)	16.30 (11.84–21.74)	0.86 (0.74–0.98)	5.92 (4.25–7.83)	6.93 (5.01–9.30)	0.68 (0.58–0.79)	4.62 (3.28–6.17)	5.26 (3.76–7.07)	0.63 (0.54–0.73)	112.72 (78.60–152.76)	128.95 (90.35–175.79)	0.65 (0.56–0.74)
Central Asia	21.11 (19.00–23.38)	22.56 (19.43–26.09)	0.29 (-0.09–0.66)	8.42 (7.57–9.31)	7.90 (6.83–9.09)	-0.17 (-0.43–0.10)	6.29 (5.64–6.97)	5.30 (4.59–6.09)	-0.56 (-0.76– -0.37)	164.87 (149.93–181.25)	132.88 (114.85–153.68)	-0.76 (-0.93– -0.58)
Central Europe	40.70 (37.69–43.89)	71.76 (63.94–79.65)	1.84 (1.67–2.01)	14.13 (13.14–15.15)	19.14 (17.16–21.14)	0.96 (0.85–1.07)	9.52 (8.86–10.16)	10.07 (9.12–11.00)	0.16 (0.08–0.23)	260.74 (243.62–278.08)	274.20 (248.91–300.59)	0.09 (-0.03–0.22)
Eastern Europe	34.96 (32.34–38.52)	57.27 (50.84–64.35)	1.36 (1.12–1.60)	15.09 (14.12–16.38)	20.29 (18.06–22.61)	0.58 (0.37–0.78)	8.67 (8.14–9.32)	8.96 (7.96–9.99)	-0.38 (-0.60– -0.16)	245.89 (231.36–265.94)	256.41 (227.19–287.04)	-0.40 (-0.64– -0.17)
High-income Asia Pacific	29.84 (26.67–33.28)	47.60 (39.57–56.01)	1.61 (1.29–1.92)	9.40 (8.47–10.34)	13.28 (10.98–15.45)	1.00 (0.68–1.33)	3.42 (3.15–3.61)	3.90 (3.31–4.30)	0.08 (-0.29– 0.45)	80.82 (75.73–85.34)	82.89 (73.01–90.73)	-0.28 (-0.66–0.10)
Australasia	116.74 (96.70–141.26)	127.27 (102.20–156.43)	0.32 (0.15–0.50)	23.99 (19.92–28.79)	22.59 (18.16–27.57)	-0.19 (-0.42–0.03)	6.35 (5.48–7.24)	4.64 (3.90–5.41)	-0.97 (-1.27– -0.67)	160.43 (138.73–184.54)	111.88 (94.64–131.03)	-1.12 (-1.41– -0.84)
Western Europe	86.23 (78.71–94.75)	95.08 (85.94–103.87)	0.45 (0.32–0.57)	19.45 (17.75–21.29)	18.53 (16.67–20.23)	-0.06 (-0.13– 0.01)	7.86 (7.25–8.43)	5.44 (4.89–5.87)	-1.15 (-1.22– -1.07)	214.54 (198.48–231.14)	136.84 (125.42–147.25)	-1.45 (-1.49– -1.40)
Southern Latin America	27.55 (23.14–32.65)	29.39 (24.71–34.58)	0.47 (0.19–0.75)	9.74 (8.30–11.37)	8.44 (7.12–9.88)	-0.20 (-0.45– 0.05)	5.95 (5.16–6.82)	4.29 (3.67–4.94)	-0.71 (-0.95– -0.48)	152.74 (132.38–175.88)	103.61 (89.18–119.49)	-0.98 (-1.23– -0.73)
High-income North America	121.27 (114.65–127.00)	105.37 (98.15–111.28)	-0.40 (-0.47– -0.32)	23.91 (22.48–25.07)	19.43 (17.89–20.58)	-0.65 (-0.75– -0.54)	6.91 (6.49–7.19)	4.69 (4.28–4.95)	-1.21 (-1.39– -1.03)	178.36 (169.98–185.30)	112.92 (105.62–118.98)	-1.43 (-1.61– -1.25)
Caribbean	37.02 (32.39–42.09)	43.04 (34.92–52.12)	0.79 (0.66–0.93)	13.81 (12.18–15.60)	13.34 (10.94–16.04)	0.12 (0.00–0.23)	9.12 (8.11–10.27)	7.74 (6.38–9.21)	-0.29 (-0.40– -0.17)	211.94 (188.01–240.42)	186.53 (152.81–223.44)	-0.18 (-0.30– -0.06)
Andean Latin America	8.95 (7.30–10.93)	15.17 (11.39–20.14)	1.91 (1.73–2.09)	4.35 (3.58–5.32)	5.12 (3.89–6.70)	0.59 (0.41–0.77)	3.48 (2.86–4.23)	3.15 (2.41–4.04)	-0.26 (-0.40– -0.12)	79.75 (65.52–96.96)	70.62 (53.68–91.32)	-0.37 (-0.52– -0.21)
Central Latin America	13.43 (12.56–14.29)	17.16 (14.87–19.58)	0.64 (0.55–0.74)	6.10 (5.70–6.45)	5.80 (5.04–6.57)	-0.36 (-0.48– -0.25)	4.64 (4.34–4.90)	3.58 (3.12–4.03)	-0.99 (-1.10–-0.88)	100.64 (94.96–105.95)	79.51 (69.55–90.09)	-0.96 (-1.07– -0.84)
Tropical Latin America	28.24 (25.91–30.70)	37.82 (34.33–41.37)	0.89 (0.80–0.98)	11.94 (10.93–12.94)	12.49 (11.27–13.64)	0.12 (0.02–0.21)	8.73 (7.99–9.43)	7.63 (6.88–8.29)	-0.40 (-0.51–-0.30)	221.05 (203.93–238.88)	194.76 (178.39–210.97)	-0.44 (-0.57– -0.31)
North Africa and Middle East	7.88 (6.60–9.28)	13.74 (11.62–16.15)	1.85 (1.77–1.93)	3.42 (2.83–4.07)	4.28 (3.62–4.99)	0.78 (0.73–0.84)	2.55 (2.10–3.04)	2.43 (2.06–2.81)	-0.12 (-0.17–-0.08)	58.87 (48.92–70.27)	54.90 (46.76–63.76)	-0.23 (-0.27– -0.20)
South Asia	50.01 (44.31–56.25)	76.42 (65.60–86.82)	1.27 (1.09–1.45)	25.35 (22.25–28.64)	30.87 (26.40–35.11)	0.49 (0.36–0.62)	20.67 (18.07–23.43)	21.65 (18.53–24.63)	-0.16 (-0.21– -0.10)	530.39 (467.52–599.12)	546.84 (467.11–623.00)	-0.03 (-0.11–0.05)
Central Sub-Saharan Africa	11.99 (8.67–16.51)	15.63 (11.08–21.24)	0.85 (0.60–1.10)	6.71 (4.86–9.27)	7.47 (5.28–10.24)	0.32 (0.15–0.48)	5.84 (4.20–8.09)	6.10 (4.25–8.46)	0.12 (0.01–0.23)	143.64 (103.73–199.59)	148.82 (105.00–204.22)	0.10 (-0.01–0.21)
Eastern Sub-Saharan Africa	19.75 (16.73–23.05)	24.16 (19.23–29.25)	0.56 (0.40–0.72)	10.81 (9.14–12.62)	11.27 (9.08–13.48)	0.01 (-0.07–0.09)	9.22 (7.81–10.78)	8.95 (7.23–10.69)	-0.20 (-0.24– -0.15)	232.10 (196.28–272.01)	221.43 (175.89–267.84)	-0.27 (-0.33– -0.22)
Southern Sub-Saharan Africa	29.18 (21.36–36.44)	33.18 (28.52–38.04)	0.47 (0.19–0.75)	12.73 (9.33–15.86)	13.44 (11.61–15.36)	0.03 (-0.13–0.19)	9.45 (6.93–11.77)	9.34 (8.10–10.64)	-0.19 (-0.44– 0.05)	249.79 (183.72–310.84)	246.05 (212.70–281.80)	-0.21 (-0.46–0.04)
Western Sub-Saharan Africa	6.47 (5.22–7.70)	9.39 (7.39–11.61)	1.20 (1.09–1.32)	3.40 (2.76–4.04)	4.38 (3.51–5.34)	0.82 (0.76–0.87)	2.84 (2.31–3.36)	3.41 (2.77–4.13)	0.61 (0.55–0.66)	70.90 (57.47–83.98)	83.70 (66.31–102.76)	0.53 (0.47–0.59)

ASIR: age-standardized incidence rate. ASPR: age-standardized prevalence rate. ASMR: age-standardized mortality rate. ASDR: age-standardized disability-adjusted life years rate. EAPC: estimated annual percentage change. UI: uncertainty interval. CI: confidence interval. LOCC: lip and oral cavity cancer.

### Regional burden

Both the ASPR and ASIR of the LOCC were greater across the five SDI regions in 2021 than in 1990, with positive EAPCs, indicating significant increases in these indicators over time. Notably, the middle-SDI region underwent the most substantial growth in both ASPR (EAPC=1.87; 95% CI: 1.73–2.01) and ASIR (EAPC=0.84; 95% CI: 0.74–0.94). The low-middle-SDI region presented the most notable growth in the ASMR (EAPC=0.19; 95% CI: 0.13–0.25) and ASDR (EAPC=0.13; 95% CI: 0.08– 0.19), whereas the low-SDI region also presented a notable increase in mortality (EAPC=0.09; 95% CI: 0.01–0.18). It should be noted that the substantial overlap in their confidence intervals suggests that the upward trend in the low-middle-SDI region is not statistically distinguishable from that in the low-SDI region. By contrast, the high-SDI, high-middle-SDI, and middle-SDI regions presented declining trends in EAPCs for both mortality rates and DALY rates (Table 1).

Across the 21 GBD regions, the three regions with the highest ASPRs in 2021 were Australasia (127.27; 95% UI: 102.20–156.43), high-income North America (105.37; 95% UI: 98.15–111.28), and Western Europe (95.08; 95% UI: 85.94–103.87). The regions with the highest ASIRs were South Asia (30.87; 95% UI: 26.40–35.11), Australasia (22.59, 95% UI: 18.16–27.57), and Eastern Europe (20.29; 95% UI: 18.06–22.61). The three regions with the highest ASMRs were South Asia (21.65; 95% UI: 18.53–24.63), Central Europe (10.07; 95% UI: 9.12–11.00), and southern Sub-Saharan Africa (9.34; 95% UI: 8.10–10.64). The regions with the highest ASDRs were South Asia (546.84; 95% UI: 467.11–623.00), Central Europe (274.20; 95% UI: 248.91–300.59), and Eastern Europe (256.41; 95% UI: 227.19–287.04). Over time, the disease burden in different GBD-defined regions generally increased, with East Asia experiencing the most notable surges in prevalence (EAPC=3.40; 95% CI: 3.24–3.56) and incidence (EAPC=1.84; 95% CI: 1.66–2.03). Oceania, on the other hand, experienced the most substantial increases in mortality (EAPC=0.63; 95% CI: 0.54–0.73) and DALYs (EAPC=0.65; 95% CI: 0.56–0.74) (Table 1).

### Country burden

**Figure 1:**
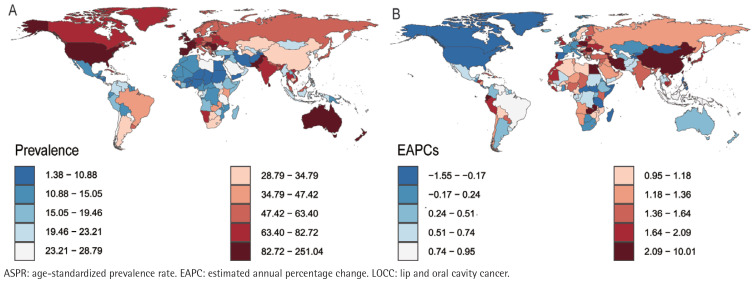
The prevalence of LOCC among adults aged ≥45 years in 204 countries and regions worldwide in 2021: A) ASPR; B) EAPC of ASPR

**Figure 2:**
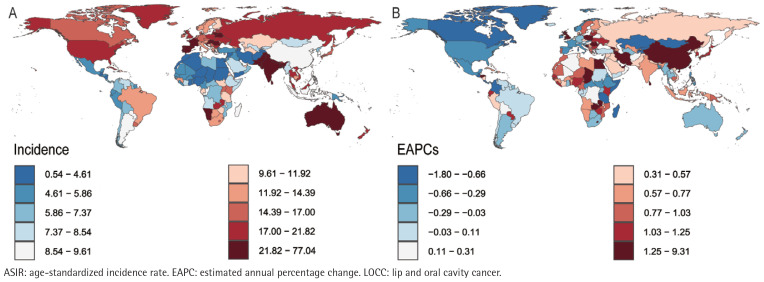
The incidence of LOCC among adults aged ≥45 years in 204 countries and regions worldwide in 2021: A) ASIR; B) EAPC of ASIR

**Figure 3:**
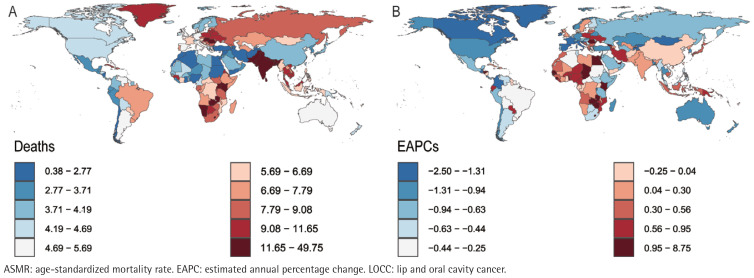
The mortality of LOCC among adults aged ≥45 years in 204 countries and regions worldwide in 2021: A) ASMR; B) EAPC of ASMR

**Figure 4:**
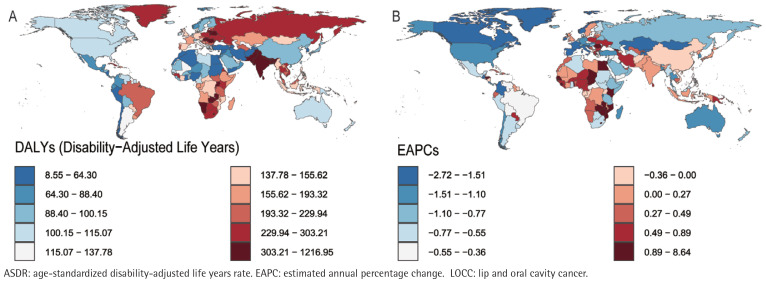
The DALYs of LOCC among adults aged ≥45 years in 204 countries and regions worldwide in 2021: A) ASDR; B) EAPC of ASDR

In 2021, the Republic of Palau presented the highest ASIR (76.27; 95% UI: 51.40– 109.87) and ASMR (49.26; 95% UI: 33.51–70.57) for LOCC among adults aged ≥45 years, closely followed by Pakistan, with an ASIR of 62.59 (95% UI: 46.14– 84.03) and an ASMR of 47.96 (95% UI: 35.71–64.24). Notably, Pakistan’s ASDR (1204.90– 95% UI: 892.71–1620.52) slightly surpassed that of Palau (1162.53; 95% UI: 775.81– 1698.43). However, the considerable overlap in their uncertainty intervals indicates that this difference is not statistically significant. Conversely, the Democratic Republic of Sao Tome and Principe recorded the lowest ASIR (1.39; 95% UI: 0.86– 2.17), ASPR (0.54; 95% UI: 0.34–0.83), ASMR (0.39; 95% UI: 0.25–0.59) and ASDR (8.63; 95% UI: 5.47–13.10) for the LOCC among individuals age ≥45 years (Figures 1A–4A).

Longitudinal temporal trend analysis revealed that the Republic of Cabo Verde demonstrated the most pronounced upward trends in ASPR (EAPC=9.91; 95% CI: 7.34–12.55), ASIR (EAPC=9.22; 95% CI: 6.60–11.91), ASMR (EAPC=8.66; 95% CI: 5.99–11.40), and ASDR (EAPC=8.55; 95% CI: 5.89–11.28). Conversely, the United States Virgin Islands demonstrated the most significant declines in ASPR (EAPC= -1.53; 95% CI: -1.78 – -1.29) and ASIR (EAPC= -1.78; 95% CI: -2.05 – -1.51), whereas the French Republic demonstrated the most notable reductions in ASMR (EAPC= -2.48; 95% CI: -2.65 – -2.31) and ASDR (EAPC= -2.69, 95% CI: -2.93 – -2.46) (Figures 1B–4B; and Supplementary file Table S1).

### Age-sex-year association analysis

The sex-age analysis indicated that for both sexes, the ASIR and ASPR of LOCC initially increased significantly but then gradually decreased with increasing age. In contrast, the ASMR for both sexes continues to increase with age. The ASDR for males first increases sharply and then levels off with minor fluctuations, whereas for females, it consistently increases and accelerates in later years. Overall, the burden indicators in males were significantly greater than in females (Supplementary file Figure 5).

The sex-time analysis revealed that from 1990 to 2021, both males and females experienced significant increases in ASIR and ASPR over time worldwide (Supplementary file Figures S1 and S2). The male ASMRs and ASDRs tended to decrease, whereas the female ASMRs and ASDRs remained stable (Supplementary file Figures S3 and S4).

The results of age-time analysis demonstrate that between 1990 and 2021, the ASPR has shown an overall upward trend in all age cohorts, with a particularly pronounced increase in the 70–74 years age cohorts. The ASIR generally increased, with the pronounced upward shift noted in the 90–94 years age cohorts (Supplementary file Figures S5 and S6). Most age groups maintained stable ASMRs, with a notable increase in mortality observed in the ≥95 years age cohorts. ASDR showed an overall declining trend, with the lowest DALY rate observed in the 45–49 years age cohorts and the highest and continuously increasing DALY rate in the ≥95 years (Supplementary file Figures S7 and S8).

Overall, joinpoint segmented regression analysis revealed that both the ASIR (AAPC=0.057; 95% CI: 0.054–0.060) and the ASPR (AAPC=0.355; 95% CI: 0.342– 0.368) exhibited significant upward trends, whereas the ASMR (AAPC= -0.005; 95% CI: -0.006 – -0.003]) and the ASDR (AAPC= -0.315; 95% CI: -0.358 – -0.271) demonstrated significant downward trends. Specifically, the trends in the ASIR and ASPR were similar, with notable inflection points occurring in 1995, 2004, and 2017 (with incidence showing an inflection in 2018) (Supplementary file: Figures 6A and 6B, and Table S2). Similarly, the trends in the ASMR and ASDR were also comparable, both initially increasing and then significantly decreasing, followed by a subsequent increase and then a decrease again. Notable inflection points occurred in 1995, 2004, and 2019. Since 2019, while the ASDR has shown a declining trend, this change has not been statistically significant (p>0.05) (Supplementary file: Figures 6C and 6D, and Table S2).

### Correlation between LOCC burden indicators and SDI

A notable correlation was identified among the ASPR, ASIR, and SDI in adults aged ≥45 years. This correlation was statistically significant (Spearman’s correlation coefficient p<0.05) and consistent across 21 GBD regions and 204 countries. Specifically, the ASIR and ASPR significantly increased with increasing SDI (Supplementary file Figure 7). The EAPC of the ASMR and ASDR decreased significantly as SDI increased (Supplementary file Figure S9). The correlation coefficient and its p-value are detailed in Supplementary file Table S3.

### Age-period-cohort analysis

Age effect analysis showed that the ASIR, ASMR, and ASDR of LOCC applied to the population aged ≥45 years gradually increased with age, whereas the ASPR initially increased but then decreased after the age of 75 years (Supplementary file Figures 8A2–8D2). A time-period effect analysis indicated that between 1990 and 2021, with the passage of time, the ASPR consistently increased (Supplementary file Figure 8A3), whereas the ASIR gradually decreased until a significant increase was observed after 2004 (Supplementary file Figure 8A4). The ASMR and ASDR initially decreased gradually but then began to rise after 2009 (Supplementary file Figures 8C3 and 8D3). A birth cohort analysis demonstrated that individuals born in later cohorts had higher ASIRs and ASPRs than those born in earlier cohorts did (Supplementary file Figures 8A4 and 8B4), whereas the ASMR and ASDR first increased but then decreased (Supplementary file Figures 8C4 and 8D4).

### Decomposition analysis of disease burden

Worldwide demographic expansion, epidemiological transitions, and aging populations have all contributed to increases in the ASIR and ASPR, with the first two factors being the primary drivers. For the ASMR and ASDR, population growth and aging populations have exacerbated the upward trend, whereas epidemiological transitions have reduced the levels of ASMR and ASDR (Supplementary file Figure 9).

Significant regional disparities exist in disease burden drivers across five SDI and 21 GBD regions. Aging substantially reduced LOCC burden in high-SDI, high-middle-SDI regions, high-income North America, Western Europe, Eastern Europe, and Central Europe. Epidemiological shifts increased ASIR and ASPR in high/high-middle-SDI regions, Western Europe, and middle-SDI regions, while decreasing ASMR and ASDR in these areas. Population growth was identified as the main factor increasing LOCC burden across all regions (Supplementary file Figure 9 and Table S4).

### Predicted trends

From 2022 to 2050, the ASIR and ASPR of LOCC in middle-aged and elderly populations are projected to continue rising, whereas the ASMR and ASDR are expected to initially decline and then increase (Supplementary file Figure 10). Specifically, it is anticipated that by 2050, the ASPR will reach 61.81 (95% UI: 37.31–86.30), and the ASIR will reach 17.09 (95% UI: 11.95–22.23). The ASMR is projected to decline from 2022 to 2040 to 7.76 (95% UI: 6.85–8.68), after which it will increase, with an estimated ASMR of 7.84 (95% UI: 5.74–9.93) by 2050. The ASDR is expected to decrease from 2022 to 2033 to 198.01 (95% UI: 183.13–212.89), followed by an increasing trend, with a projected ASDR of 205.64 (95% UI: 134.46–276.82) by 2050 (Supplementary file Table S5).

### Attributable risk

Currently, tobacco and alcohol use are significantly associated with mortality from LOCC among adults aged ≥45 years. In high- and high-middle SDI regions, alcohol consumption and smoking have been identified as the primary risk factors, though their contributions have declined over time. In middle-SDI regions, both alcohol use and chewing tobacco exhibit increasing trends in LOCC mortality. Conversely, in low-middle and low-SDI regions, chewing tobacco remains the dominant risk factor with a persistent upward trajectory, while alcohol use, though proportionally lower, demonstrates a year-on-year increase (Supplementary file: Figure 11 and Table S6).

## DISCUSSION

LOCC, a common malignant neoplasm within the spectrum of head and neck squamous cell carcinoma, poses a formidable challenge to global public health. It not only diminishes the quality of patients’ life but also imposes a considerable economic strain on society^2^. This study used GBD 2021 data to analyze the ASIR, ASPR, ASMR and ASDR of the LOCC in adults aged ≥45 years. Moreover, the impacts of various countries/regions, social demographic indices, GBD regions, gender and age groups on the burden of LOCC were studied, the related risk factors that aggravate the burden of disease were explored, and the disease burden of LOCC in adults aged ≥45 years from 2022 to 2050 was predicted. This provides important data for a holistic evaluation of the impact of LOCCs in middle-aged and elderly people on the global health system and socioeconomic development.

Our analysis of the worldwide burden of oral cancer among adults aged ≥45 years revealed that the incidence rate and prevalence of LOCC increased at average annual rates of 0.35% and 0.77%, respectively, whereas mortality and DALY tended to decrease (-0.15% and -0.25%, respectively) between 1990 and 2021. Although the current data indicate a slow decline in the ASMR and ASDR, the BAPC model prediction results show a trend characterized by an initial decrease followed by an increase. This trend indicates an intensification of public health challenges, requiring early planning of response strategies and emphasizing the importance of early prevention and health education.

From the perspective of regional differences, the ASPR and ASIR of all five SDI partitions show an increasing trend, with the medium-SDI region experiencing the fastest growth rate. The high-SDI and medium-SDI regions have experienced decreases in the ASMR and ASDR, whereas these two indicators are still increasing in the medium-low- and low-SDI regions. In areas with high SDI and medium-high SDI, the incidence rate of LOCC continues to increase. The important reasons may be the worsening of population aging^12^ and carcinogenic HPV infection^15^. Overall, countries with elevated SDI scores typically possess sufficient medical resources, advanced cancer screening and diagnosis methods^16^, high-quality cancer care^17^, high public health awareness, and effective health promotion support^18^. The risk factors for smoking and alcohol abuse in the region have significantly decreased, and relatively prosperous economic conditions and other factors have contributed to the decreases in the mortality and DALY rates of LOCC. The most pronounced change in the growth of LOCC is observed in the central SDI region, potentially driven by the accelerated pace of urbanization and industrialization in middle-income countries in recent years, which has prompted shifts in lifestyle patterns^19^. The economic development level of low-SDI and low- to medium-SDI areas is relatively low, the medical system is relatively backward, the social security level is insufficient^20^, there is a lack of professional medical personnel, insufficient equipment and technical strength, and the health awareness of residents is generally low^21,22^. The effectiveness of medical intervention measures is poor, which in turn affects the treatment effect and patient survival rate. Our research also discovered a significant correlation between SDI and the disease burden indicators of LOCC in adults aged ≥45 years. Specifically, the ASIR and ASPR increased significantly with higher SDI values, while the EAPC of the ASMR and ASDR decreased significantly as SDI increased. Therefore, while maintaining results in high-SDI and medium-high-SDI regions, targeted interventions for specific populations will be particularly important. Medium-SDI regions should do a good job in disease prevention and control while developing rapidly and focus on both aspects. Improving the distribution of medical resources and refining public health policies in low-income and low-middle-income regions could potentially alleviate the burden of oral cancer in these areas.

In 21 GBD regions, we found that in 2021, the three indicators (ASIR, ASMR and ASDR) of the LOCC in adults aged ≥45 years were the highest in South Asia, whereas the ASPR was the highest in Australia. In terms of time, the prevalence rate (3.40%) and incidence rate (1.84%) in East Asia increased the most, whereas the mortality rate (0.63%) and DALYs (0.65%) in Oceania increased the most significantly. In regions such as South Asia, the burden of oral cancer is disproportionately high, which is closely related to lifestyle factors such as tobacco and betel nut juice use^23,24^. Southeast Asian countries, especially in areas lacking effective screening and treatment resources, still have a heavy burden of oral cancer^25^. In Australia, the incidence rate of LOCC increased by 1.3 times between 1999 and 2018, indicating an increasing trend of cancer burden in the region^26^. According to one study, the annual medical expenses for oral cancer in Australia in 2019 were approximately AUD 113.2 million, with an overall economic burden of AUD 2.1 billion^27^. These data are consistent with our findings that lip and oral cancer not only have a serious impact on patients’ health but also pose challenges to the country’s healthcare system and economic development.

Our results from the national level analysis show that in 2021, Palau and Pakistan had the heaviest disease burdens, whereas Sao Tome and Principe had the lowest disease burdens. The vertical trend shows that Cape Verde has the most significant deterioration in various indicators, while the US Virgin Islands (ASPR= -1.53%, ASIR= -1.78%) and France (ASMR= -2.48%, ASDR= -2.69%) have achieved the most significant improvement. Palau also has a long history of chewing tobacco^28^, whereas Pakistan has a high usage rate of tobacco and betel nut^29^, which is the main reason for the severe disease burden. Under the 2018–2022 National Health Strategy, the French government has boosted healthcare funding by allocating €400 million over five years to support preventive health programs and per capita health investments^30^. Despite smoking and alcohol consumption rates remaining above the EU average, stringent preventive legislation and its rigorous enforcement have yielded positive outcomes. Our findings also demonstrate a notable reduction in the burden of LOCC in France.

Our research revealed that from 1990 to 2021, the ASIR and ASPR of adult males and females aged ≥45 years significantly increased over time worldwide. Both the male ASMR and ASDR tended to decrease, whereas the female ASMR and ASDR remained stable, with a much greater disease burden in males than in females. From 1990 to 2021, the prevalence rate of the 70–74 years age group increased significantly, the incidence rate of the 90–94 years age group increased most significantly, the mortality rate of the ≥95 years age group increased significantly, and the ASDR overall showed a downward trend; however, the DALY rate of the ≥95 years age group was the highest and continued to rise. The greater the age is, the greater the disease burden. The age cohort effect analysis demonstrated that the ASIR, ASMR and ASDR of ≥45 years adults with LOCC gradually increased with age. The ASIR and ASPR of individuals born later are greater than those of individuals born earlier. These results collectively reveal the comprehensive influence of age, time, and historical factors on the occurrence and development of LOCC disease, providing a comprehensive perspective for understanding the dynamic trends of the disease.

At both global and national scales, there remains a significant knowledge gap regarding the distribution and trends of risk factors linked to oral cancer. Hence, it is crucial to study the co-occurrence of oral cancer and its associated risk factors to generate robust evidence for informing primary prevention strategies against oral cancer. According to the GBD 2021 framework, behavioral risk factors (Level 1) encompass tobacco and alcohol use, with tobacco further categorized as a Level 2 risk factor comprising smoking and chewing tobacco^31^. This study conducted an in-depth exploration of these risk factors.

Since 2003, the World Health Organization Framework Convention on Tobacco Control has secured ratification from 182 contracting parties, which collaborate under WHO leadership to reduce tobacco consumption through measures such as implementing smoking bans and graphic health warnings, raising tobacco taxes and prohibiting tobacco advertising^32^. After more than a decade of effort, people have become increasingly aware that smoking is harmful to health, and our research confirms a concurrent decline in smoking prevalence both worldwide and across various SDI regions.

However, this study reveals that in low-middle- and low-SDI regions, chewing tobacco remains the dominant risk factor for oral cancer, with a persistent upward trajectory. In Asian countries such as Pakistan, India, Sri Lanka, Bangladesh, and Indonesia, betel quid chewing – often combined with tobacco – constitutes a prevalent high-risk habit. Multiple studies have documented its dose-dependent association with increased oral cancer risk^33^. An investigation among tobacco users demonstrated a 13-fold elevation in oral lesion risk among chewers compared to non-users^34^. Establishing optimal oral hygiene practices and adopting a wholesome lifestyle are recognized as critical health behaviors contributing to overall physical and mental well-being. However, policymakers must devise strategies to overcome political and economic barriers that hinder efforts to reduce tobacco consumption prevalence.

Moreover, alcohol consumption remains a global risk factor, with particularly high prevalence in high- and high-middle SDI regions. Meanwhile, other regions have witnessed a rising trend in alcohol use. Research has shown that strong liquor exerts a significantly greater influence on the risk of oral cancer compared to low-alcohol beverages such as beer and wine^35^.A meta-analysis found that each incremental 10 g of daily alcohol intake raises the risk of oral and pharyngeal cancer by approximately 9%^36^. Therefore, further efforts are warranted to investigate effective strategies for curbing alcohol consumption, thereby reducing the disease burden and mortality associated with oral cancer.

### Limitations

This study relies on the GBD database, which covers data from only 1990–2021. Data updates in the GBD database may be delayed, and the latest epidemiological data may not be included. Although the study covers data from global, regional, and national scales, there may be insufficient data for certain regions or specific populations, which could affect a thorough understanding of the global distribution characteristics of the LOCC. The prediction of future trends is based on existing data and models, which may be limited by model assumptions and parameter selection. The accuracy of the prediction results needs to be verified with future data. In addition, our analysis focused on estimating independent trends for various population subgroups using EAPC. While these metrics robustly describe the temporal pattern within each group, our study was not designed to perform formal statistical tests (e.g. via interaction terms or z-tests) for the differences in trends between groups. Future research with a specific hypothesis-testing framework could further elucidate the comparative dynamics of LOCC burden across different demographics and regions. Although this study has certain limitations, it provides epidemiological data on LOCCs in adults aged ≥45 years on a global scale, which helps global health policy makers and public health experts understand the distribution characteristics and development trends of LOCC in middle-aged and elderly people and formulate enhanced prevention and control strategies on the basis of the shift in the global population distribution toward aging.

## CONCLUSIONS

The incidence and prevalence of LOCC in ≥45 years adults worldwide have been continuously increasing, and the overall disease burden of LOCC remains severe, with significantly more males than females and significant regional and national differences that cannot be ignored. Although the current data show a decline in the ASMR and ASDR, future predictions estimate that both will show an initial decline followed by a subsequent increase. Therefore, particular emphasis should be placed on the middle-aged and elderly male population and high-risk areas to achieve early prevention and reduce the disease burden of LOCC. In addition, the multifactorial driving characteristics of LOCC require comprehensive consideration of multiple factors in public health interventions to achieve more effective disease management and control.

## Supplementary Material



## Data Availability

The data supporting this research are available from the authors on reasonable request.

## References

[1] Almangush A, Pirinen M, Youssef O, Mäkitie AA, Leivo I. Risk stratification in oral squamous cell carcinoma using staging of the eighth American Joint Committee on Cancer: systematic review and meta-analysis. Head Neck. 2020;42(10):3002-3017. doi:10.1002/hed.26344 32548858

[2] Bray F, Laversanne M, Sung H, et al. Global cancer statistics 2022: GLOBOCAN estimates of incidence and mortality worldwide for 36 cancers in 185 countries. CA Cancer J Clin. 2024;74(3):229-263. doi:10.3322/caac.21834 38572751

[3] Nokovitch L, Maquet C, Crampon F, et al. Oral cavity squamous cell carcinoma risk factors: state of the art. J Clin Med. 2023;12(9):3264. doi:10.3390/jcm12093264 37176704 PMC10179259

[4] Boffetta P, Hecht S, Gray N, Gupta P, Straif K. Smokeless tobacco and cancer. Lancet Oncol. 2008;9(7):667-675. doi:10.1016/S1470-2045(08)70173-6 18598931

[5] Agents Classified by the IARC Monographs, Volumes 1–139. The International Agency for Research on Cancer. Accessed October 13, 2025. https://monographs.iarc.who.int/agents-classified-by-the-iarc/

[6] Secretan B, Straif K, Baan R, et al; WHO International Agency for Research on Cancer Monograph Working Group. A review of human carcinogens—part E: tobacco, areca nut, alcohol, coal smoke, and salted fish. Lancet Oncol. 2009;10(11):1033-1034. doi:10.1016/s1470-2045(09)70326-2 19891056

[7] Guha N, Warnakulasuriya S, Vlaanderen J, Straif K. Betel quid chewing and the risk of oral and oropharyngeal cancers: a meta-analysis with implications for cancer control. Int J Cancer. 2014;135(6):1433-1443. doi:10.1002/ijc.28643 24302487

[8] Hecht SS, Hatsukami DK. Smokeless tobacco and cigarette smoking: chemical mechanisms and cancer prevention. Nat Rev Cancer. 2022;22(3):143-155. doi:10.1038/s41568-021-00423-4 34980891 PMC9308447

[9] Zhu S, Zhang F, Zhao G, et al. Trends in the global burden of oral cancer joint with attributable risk factors: results from the global burden of disease study 2019. Oral Oncol. 2022;134:106189. doi:10.1016/j.oraloncology.2022.106189 36208599

[10] Yang Y, Zhou M, Zeng X, Wang C. The burden of oral cancer in China, 1990-2017: an analysis for the Global Burden of Disease, Injuries, and Risk Factors Study 2017. BMC Oral Health. 2021;21(1):44. doi:10.1186/s12903-020-01386-y 33509185 PMC7842005

[11] Sargeran K, Murtomaa H, Safavi SM, Vehkalahti M, Teronen O. Malignant oral tumors in Iran: ten-year analysis on patient and tumor characteristics of 1042 patients in Tehran. J Craniofac Surg. 2006;17(6):1230-1233. doi:10.1097/01.scs.0000246728.23483.ce 17119436

[12] Ageing and health. World Health Organization. Accessed October 13, 2025. https://www.who.int/news-room/fact-sheets/detail/ageing-and-health

[13] GBD 2021 Diseases and Injuries Collaborators. Global incidence, prevalence, years lived with disability (YLDs), disability-adjusted life-years (DALYs), and healthy life expectancy (HALE) for 371 diseases and injuries in 204 countries and territories and 811 subnational locations, 1990-2021: a systematic analysis for the Global Burden of Disease Study 2021. Lancet. 2024;403(10440):2133-2161. doi:10.1016/S0140-6736(24)00757-8 38642570 PMC11122111

[14] GBD 2021 Causes of Death Collaborators. Global burden of 288 causes of death and life expectancy decomposition in 204 countries and territories and 811 subnational locations, 1990-2021: a systematic analysis for the Global Burden of Disease Study 2021. Lancet. 2024;403(10440):2100-2132. doi:10.1016/S0140-6736(24)00367-2 38582094 PMC11126520

[15] Anantharaman D, Abedi-Ardekani B, Beachler DC, et al. Geographic heterogeneity in the prevalence of human papillomavirus in head and neck cancer. Int J Cancer. 2017;140(9):1968-1975. doi:10.1002/ijc.30608 28108990 PMC8969079

[16] Zabala A, Martín-Arregui FJ, Sagazola J, Santaolalla FJ, Santaolalla F. An evaluation of an innovative screening program based on risk criteria for early diagnosis of head and neck cancers. Front Public Health. 2023;10:1004039. doi:10.3389/fpubh.2022.1004039 36699893 PMC9868380

[17] Sofi-Mahmudi A, Masinaei M, Shamsoddin E, et al. Global, regional, and national burden and quality of care index (QCI) of lip and oral cavity cancer: a systematic analysis of the Global Burden of Disease Study 1990–2017. BMC Oral Health. 2021;21(1):558. doi:10.1186/s12903-021-01918-0 34724951 PMC8561915

[18] Wu J, Chen H, Liu Y, Yang R, An N. The global, regional, and national burden of oral cancer, 1990–2021: a systematic analysis for the Global Burden of Disease Study 2021. J Cancer Res Clin Oncol. 2025;151(2):53. doi:10.1007/s00432-025-06098-w 39875744 PMC11775039

[19] Petti S. Lifestyle risk factors for oral cancer. Oral Oncol. 2009;45(4-5):340-350. doi:10.1016/j.oraloncology.2008.05.018 18674956

[20] Ramsey T, Guo E, Svider PF, et al. Laryngeal cancer: global socioeconomic trends in disease burden and smoking habits. Laryngoscope. 2018;128(9):2039-2053. doi:10.1002/lary.27068 29508408

[21] Herrera-Serna BY, Lara-Carrillo E, Toral-Rizo VH, Cristina do Amaral R, Aguilera-Eguía RA. Relationship between the Human Development Index and its components with oral cancer in Latin America. J Epidemiol Glob Health. 2019;9(4):223-232. doi:10.2991/jegh.k.191105.001 31854163 PMC7310789

[22] Attar E, Dey S, Hablas A, et al. Head and neck cancer in a developing country: a population-based perspective across 8 years. Oral Oncol. 2010;46(8):591-596. doi:10.1016/j.oraloncology.2010.05.002 20619719 PMC3223856

[23] Mathunny MMS, Sivakumar R, Padmakumar SK. A comparative analysis of the burden of lip and oral cavity cancers in the Indian subcontinent. J Oral Maxillofac Pathol. 2024;28(4):565-569. doi:10.4103/jomfp.jomfp_109_24 39949697 PMC11819645

[24] Ren ZH, Hu CY, He HR, Li YJ, Lyu J. Global and regional burdens of oral cancer from 1990 to 2017: results from the global burden of disease study. Cancer Commun (Lond). 2020;40(2-3):81-92. doi:10.1002/cac2.12009 32067418 PMC7163731

[25] Alshami ML, Al-Maliky MA, Alsagban AA, Alshaeli AJ. Epidemiology and incidence of oral squamous cell carcinoma in the Iraqi population over 5 years (2014–2018). Health Sci Rep. 2023;6(4):e1205. doi:10.1002/hsr2.1205 37064317 PMC10090270

[26] Sethi S, Ju X, Logan R, Hedges J, Garvey G, Jamieson L. Lip, oral and oropharyngeal cancer incidence among Aboriginal and Torres Strait Islander Peoples: first report from Australian population-based cancer registry, 1999–2018. Aust Dent J. 2024;69(3):182-188. doi:10.1111/adj.13013 38469883

[27] Gama MAB, Tonmukayakul U, Saraswat N, McCaffrey N, Nguyen TM. Cost of illness study on oral cancer in Australia. Oral Dis. 2025;31(7):2160-2166. doi:10.1111/odi.15267 39846356 PMC12319361

[28] Rieth K, Sy A, McIntosh S, et al. Dental health utilization in Palau: feasibility of an oral cancer screening program. Ann Glob Health. 2023;89(1):60. doi:10.5334/aogh.4174 37745775 PMC10516142

[29] Qureshi MA, Syed SA, Sharafat S. Lip and oral cavity cancers (C00–C06) from a mega city of Pakistan: ten-year data from the Dow Cancer Registry. J Taibah Univ Med Sci. 2021;16(4):624-627. doi:10.1016/j.jtumed.2021.02.001 34408617 PMC8348287

[30] France: Country Health Profile 2019. Organisation for Economic Co-operation and Development. Accessed October 13, 2025. https://www.oecd-ilibrary.org/social-issues-migration-health/france-country-health-profile-2019_d74dbbda-en

[31] GBD 2021 Risk Factors Collaborators. Global burden and strength of evidence for 88 risk factors in 204 countries and 811 subnational locations, 1990–2021: a systematic analysis for the Global Burden of Disease Study 2021. Lancet. 2024;403(10440):2162-2203. doi:10.1016/S0140-6736(24)00933-4 38762324 PMC11120204

[32] World Health Organization. WHO Framework Convention on Tobacco Control. 2003. Accessed October 13, 2025. http://apps.who.int/iris/bitstream/10665/42811/1/9241591013.pdf?ua=1

[33] Amtha R, Razak IA, Basuki B, et al. Tobacco (kretek) smoking, betel quid chewing, and risk of oral cancer in a selected Jakarta population. Asian Pac J Cancer Prev. 2014;15(20):8673-8678. doi:10.7314/apjcp.2014.15.20.8673 25374188

[34] Al-Attas SA, Ibrahim SS, Amer HA, Darwish Zel-S, Hassan MH. Prevalence of potentially malignant oral mucosal lesions among tobacco users in Jeddah, Saudi Arabia. Asian Pac J Cancer Prev. 2014;15(2):757-762. doi:10.7314/apjcp.2014.15.2.757 24568491

[35] Petti S, Scully C. Oral cancer: the association between nation-based alcohol-drinking profiles and oral cancer mortality. Oral Oncol. 2005;41(8):828-834. doi:10.1016/j.oraloncology.2005.04.004 15979385

[36] Bagnardi V, Rota M, Botteri E, et al. Alcohol consumption and site-specific cancer risk: a comprehensive dose-response meta-analysis. Br J Cancer. 2015;112(3):580-593. doi:10.1038/bjc.2014.579 25422909 PMC4453639

