# Supplementary material for: Global burden of lip and oral cavity cancer across adults aged ≥45 years from 1990 to 2021 and projections to 2050
Source: Tob Induc Dis. 2025 Dec 10;23:10.18332/tid/211972. doi: 10.18332/tid/211972 (PMC12691342; doi:10.18332/tid/211972)
Supplement: Supplementary file 1 [file TID-23-192-s1.pdf]

Figure 1-11 captions

Figure 1. The prevalence of LOCC among adults aged ≥45 years in 204 countries and regions worldwide in 2021: A) ASPR; B) EAPC of ASPR

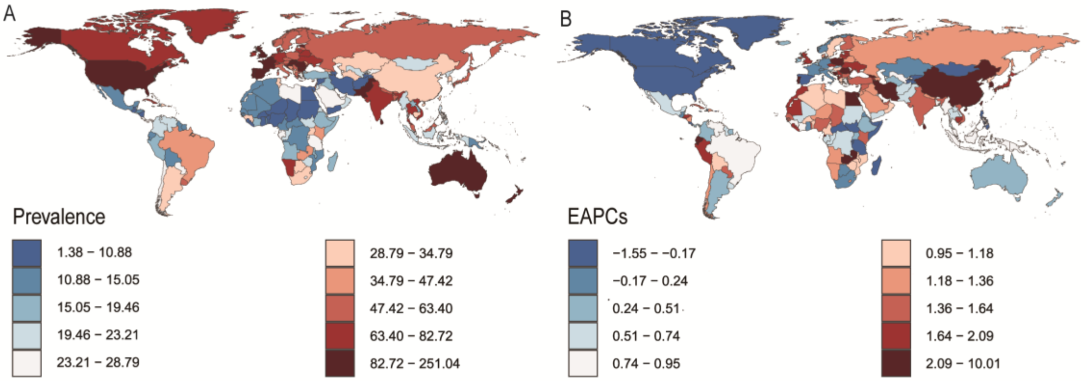

ASPR: Age-standardized prevalence rate. EAPC: Estimated annual percentage change.LOCC: lip and oral cavity cancer.

Figure 2. The incidence of LOCC among adults aged ≥45 years in 204 countries and regions worldwide in 2021: A) ASIR; B) EAPC of ASIR

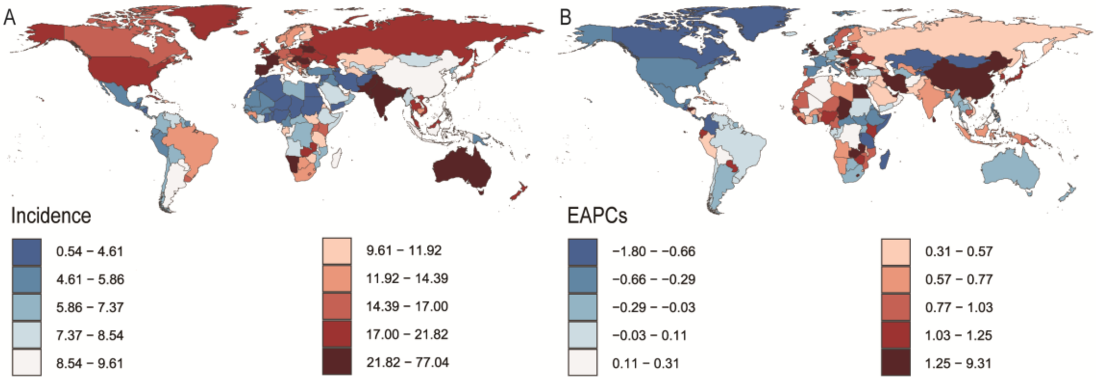

ASIR: Age-standardized incidence rate. EAPC: Estimated annual percentage change. LOCC: lip and oral cavity cancer.

Figure 3: The mortality of LOCC among adults aged ≥45 years in 204 countries and regions worldwide in 2021: A) ASMR; B) EAPC of ASMR

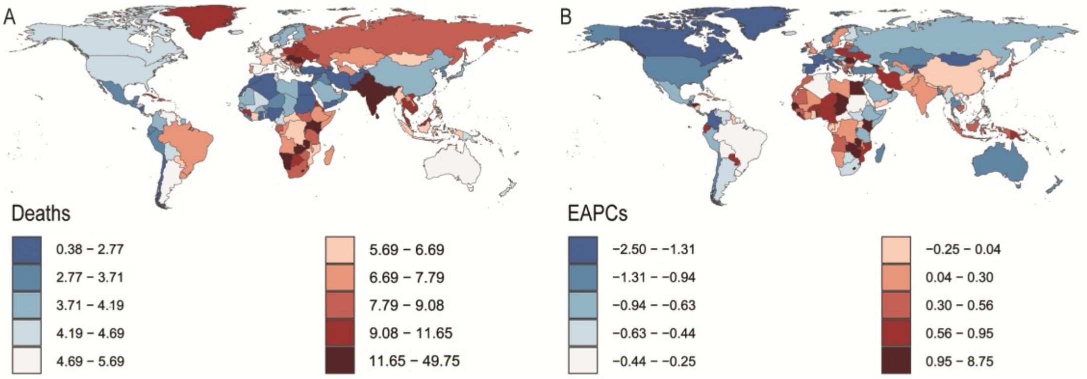

ASMR: Age-standardized mortality rate. EAPC: Estimated annual percentage change. LOCC: lip and oral cavity cancer.

Figure 4: The DALYs of LOCC among adults aged ≥45 years in 204 countries and regions worldwide in 2021: A) ASDR; B) EAPC of ASDR

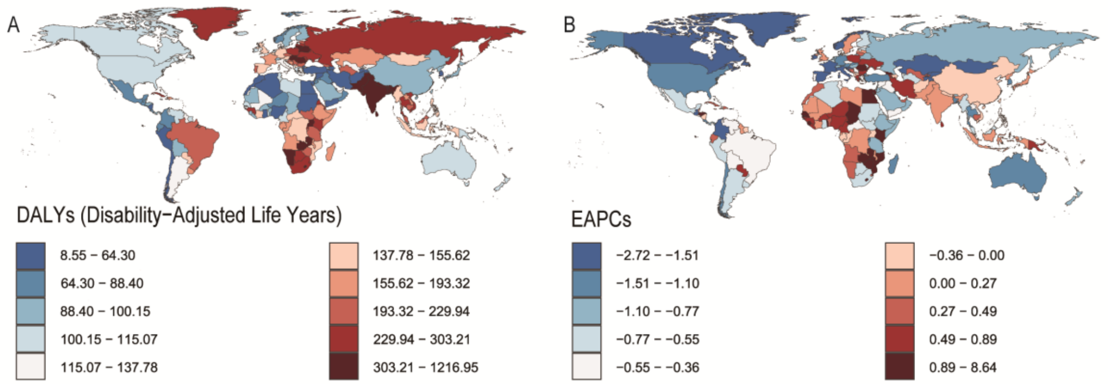

ASDR: Age-standardized disability-adjusted life years rate. EAPC: Estimated annual percentage change. LOCC: lip and oral cavity cancer.

Figure 5: Age and sex differences in ASR of LOCC among adults aged 45 and above（A.ASPR, B.ASIR, C. ASMR, D.ASDR）

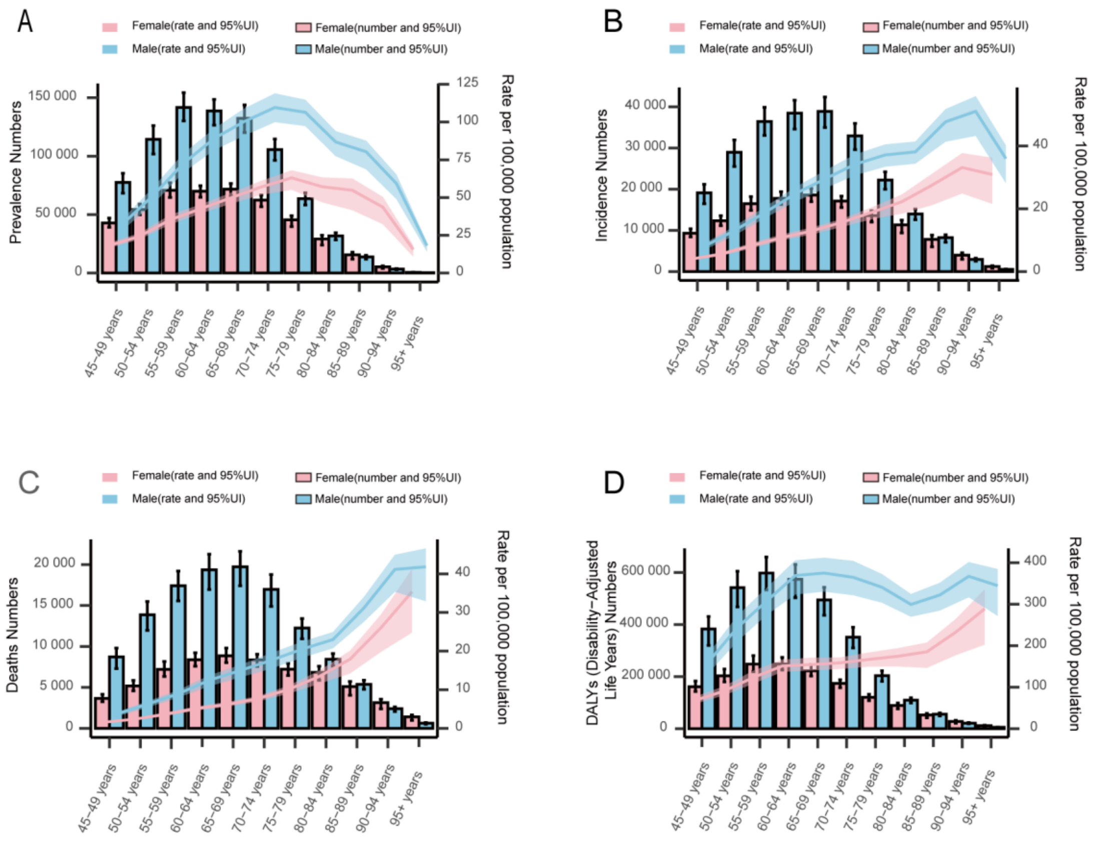

Error bars represent the 95% uncertainty intervals.The shaded areas represent the 95% uncertainty intervals of the predicted trends.ASIR: Age-standardized incidence rate. ASPR: Age-standardized prevalence rate. ASMR: Age-standardized mortality rate. ASDR: Age-standardized disability-adjusted life years rate.

Figure 6: Jointpoint analysis of LOCC disease burden in adults aged 45 and above from 1990 to 2021(A. ASPR; B. ASIR; C. ASMR; D. ASDR)

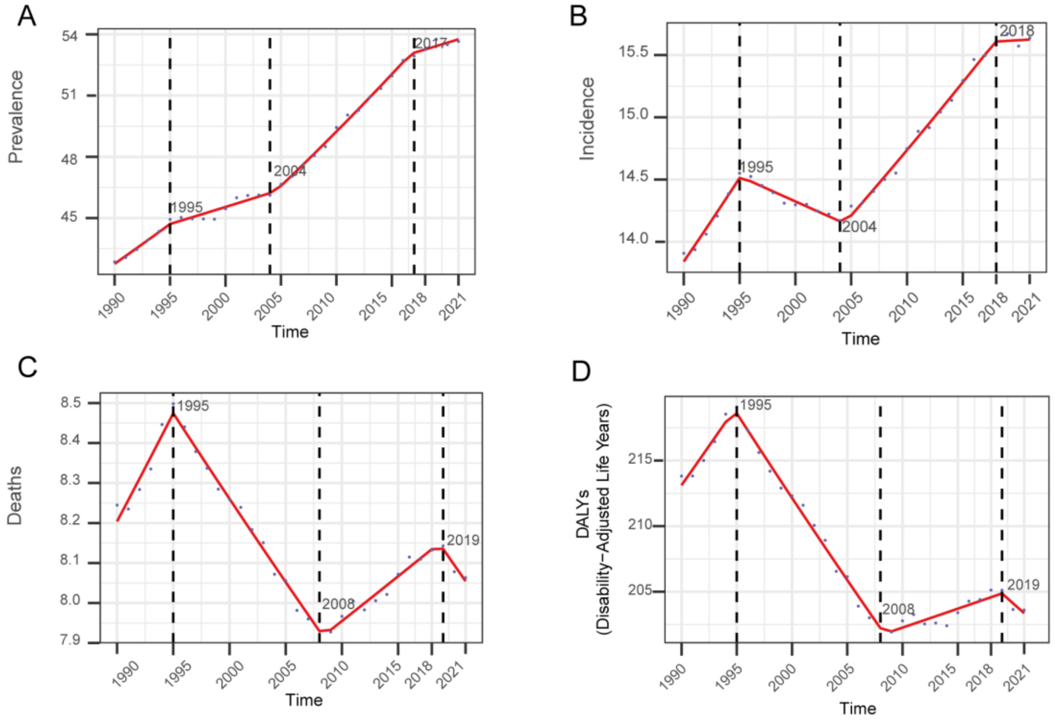

ASIR: Age-standardized incidence rate. ASPR: Age-standardized prevalence rate. ASMR: Age-standardized mortality rate. ASDR: Age-standardized disability-adjusted life years rate.

Figure 7: Correlation between ASR of LOCC and SDI in adults aged 45 and above across 21 GBD regions and 204 countries(A. ASPR in 21 regions, B. ASIR in 21 regions, C. ASPR in 204 countries, D. ASIRs in 204 countries)

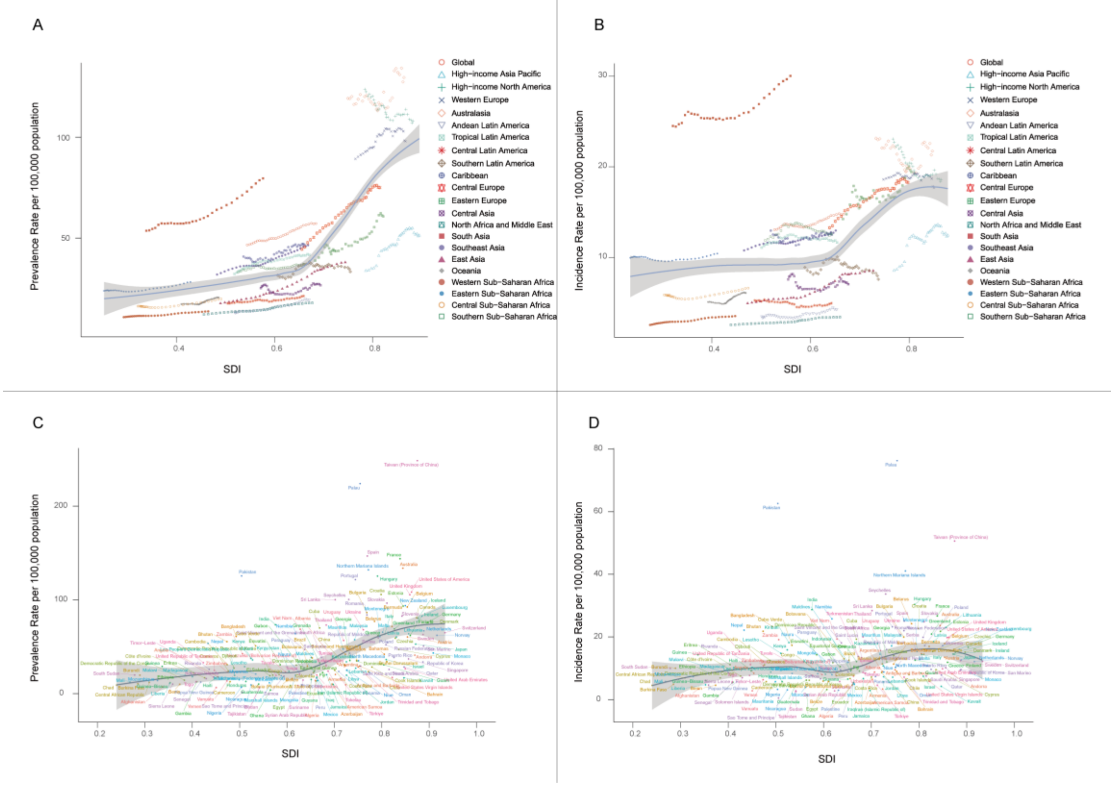

ASIR: Age-standardized incidence rate. ASPR: Age-standardized prevalence rate. ASMR: Age-standardized mortality rate. ASDR: Age-standardized disability-adjusted life years rate. SDI:sociodemographic index.

Figure 8: the APC results of LOCC disease burden in adults aged 45 and above (A.APC of ASPR,B. APC of ASIR, C. APC of ASMR, D. APC of ASDR)

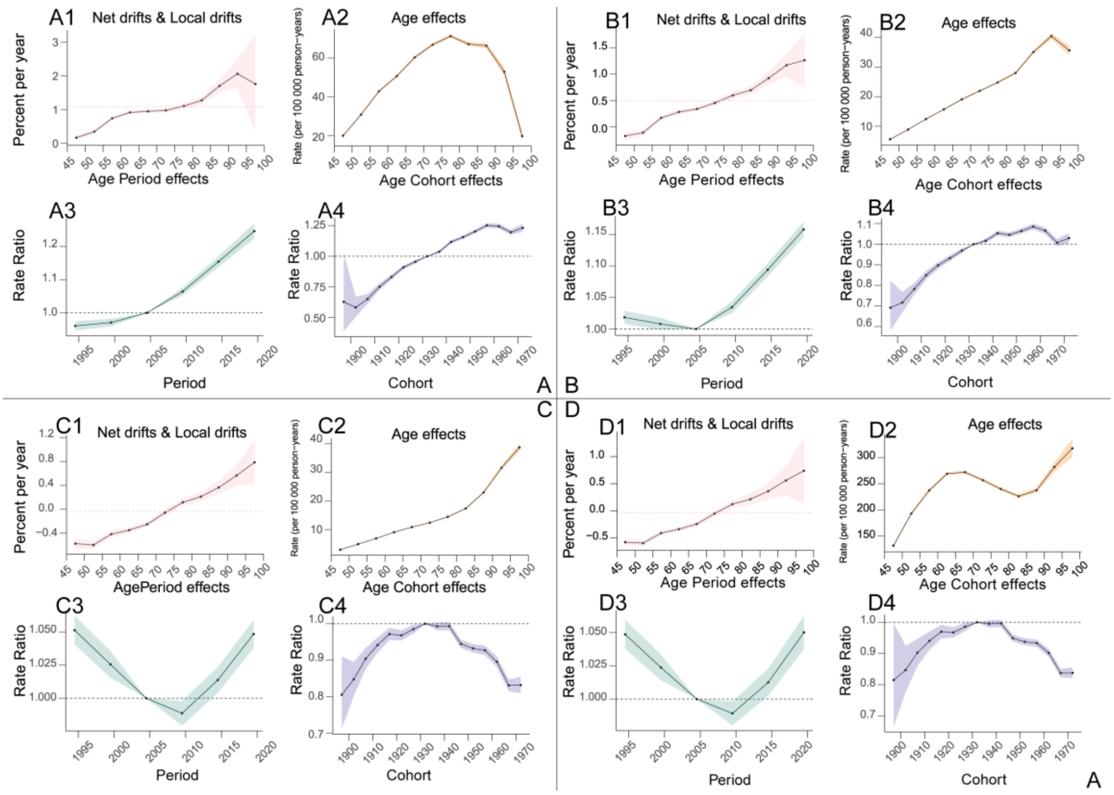

ASIR: Age-standardized incidence rate. ASPR: Age-standardized prevalence rate. ASMR: Age-standardized mortality rate. ASDR: Age-standardized disability-adjusted life years rate.APC: age-period-cohort.

Figure 9: Decomposition analysis results of LOCC disease burden in adults aged 45 and above (A. ASPR; B. ASIR; C. ASMR; ASDR)

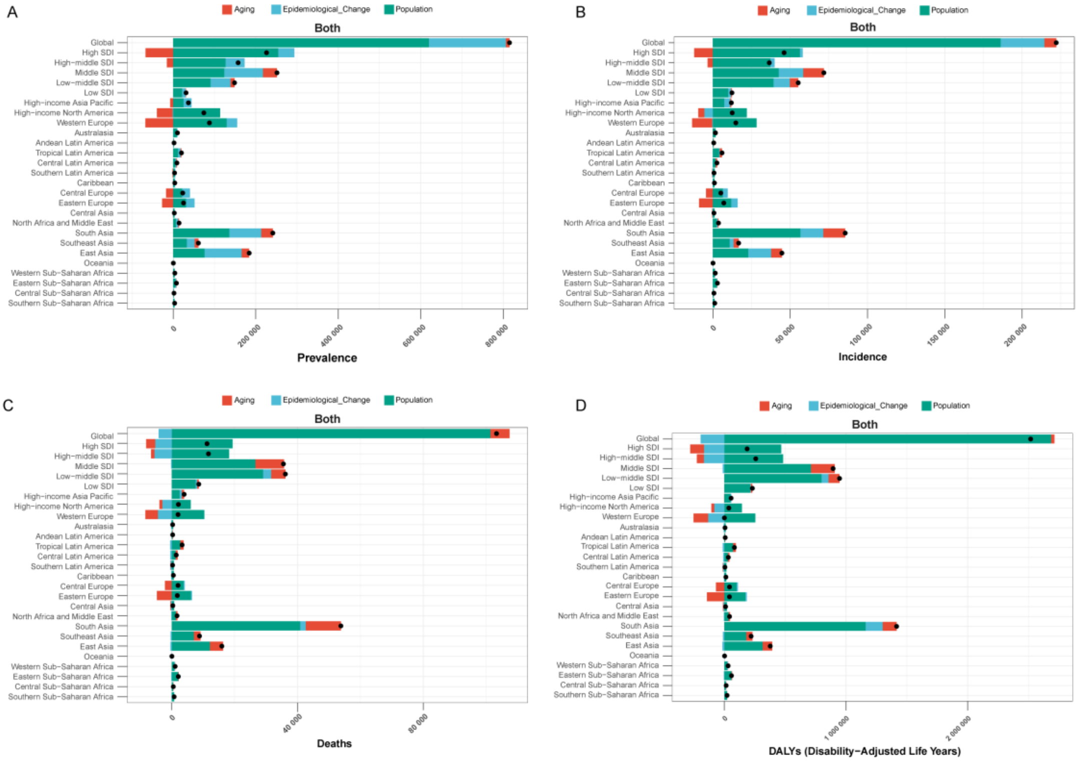

ASIR: Age-standardized incidence rate. ASPR: Age-standardized prevalence rate. ASMR: Age-standardized mortality rate. ASDR: Age-standardized disability-adjusted life years rate.

Figure 10: BAPC results of LOCC disease burden among adults aged 45 and older(A.ASPR; B. ASIR; C.ASMR;D. ASDR)

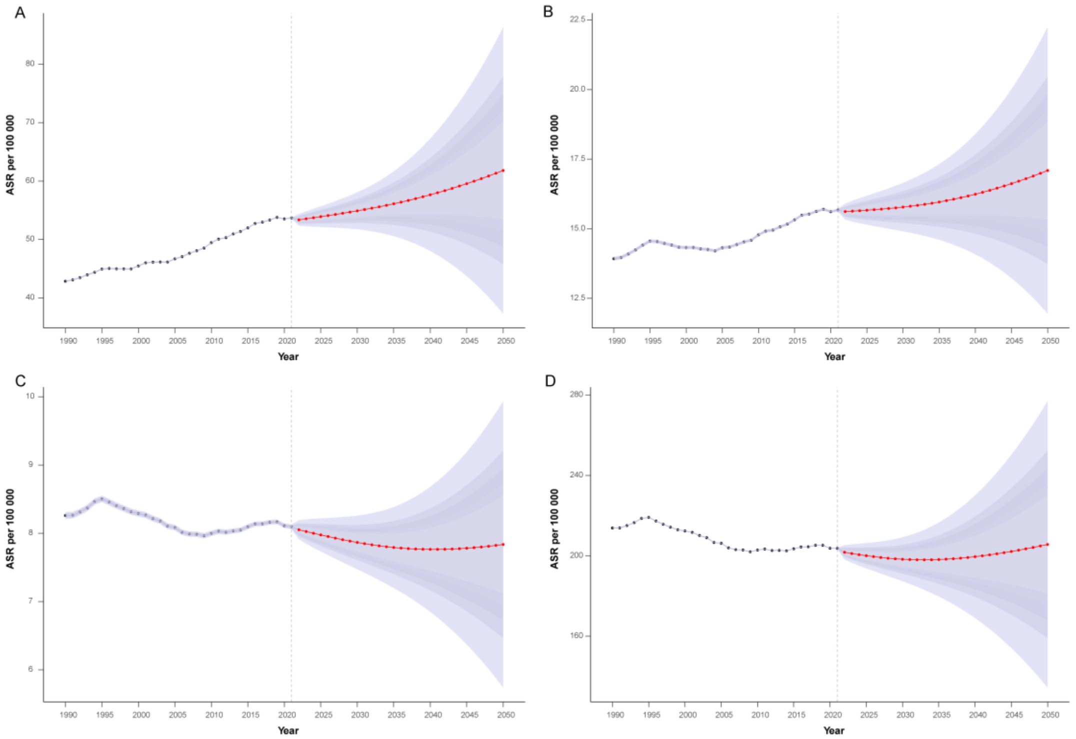

The shaded areas represent the 95% uncertainty intervals of the predicted trends. BAPC:Bayesian Age-Period-Cohort. ASIR: Age-standardized incidence rate. ASPR: Age-standardized prevalence rate. ASMR: Age-standardized mortality rate. ASDR: Age-standardized disability-adjusted life years rate.

Figure 11: Attributable Risk Factors for ASMR of LOCC in Global and Five SDI Regions (A. Attributable Risk Factors in 2021; B. Trends of Attributable Risk Factors Over Time)

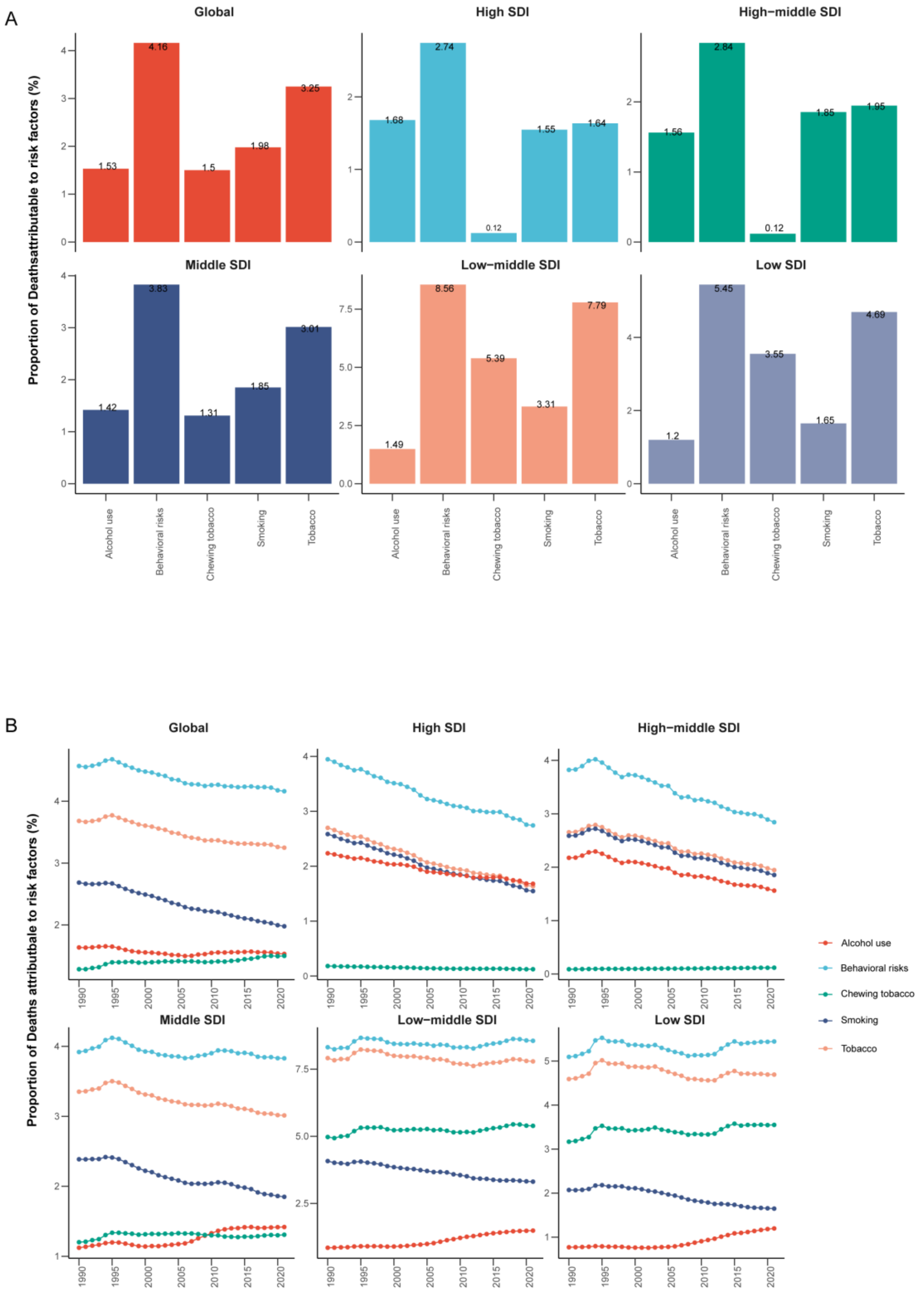

ASMR: Age-standardized mortality rate. SDI:sociodemographic index.

Figure S1-S9 captions

Figure S1: Trends in ASPR by gender for LOCC in Global and Five SDI Regions from 1990 to 2021.

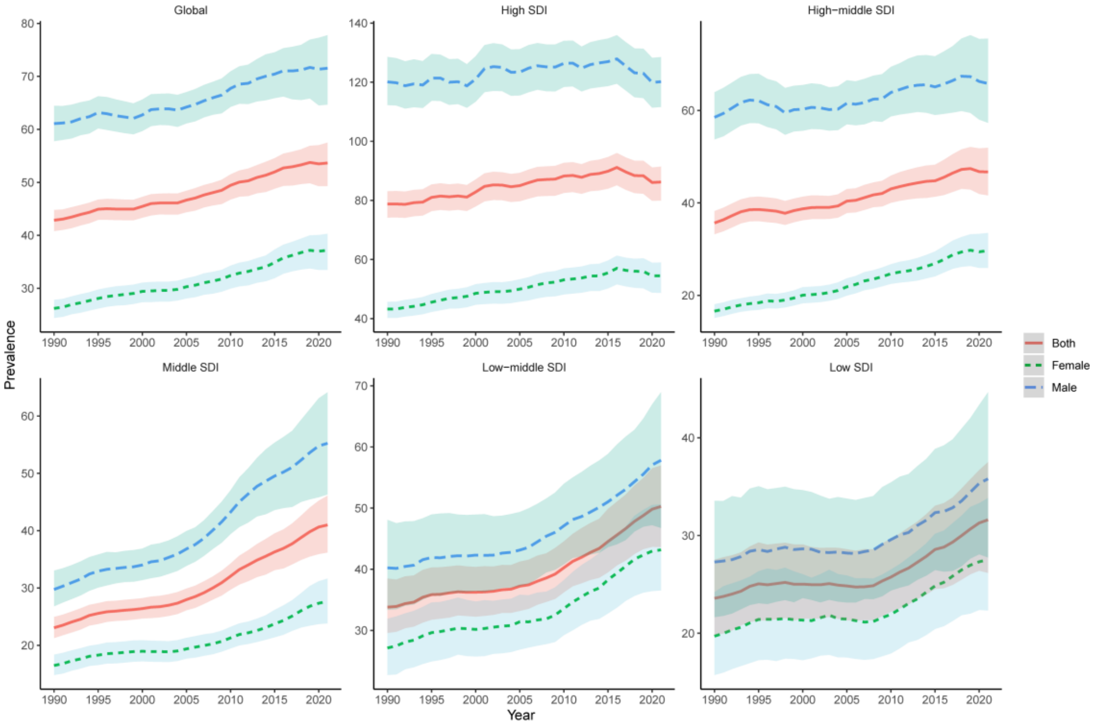

Figure S2: Trends in ASIR by gender for LOCC in Global and Five SDI Regions from 1990 to 2021

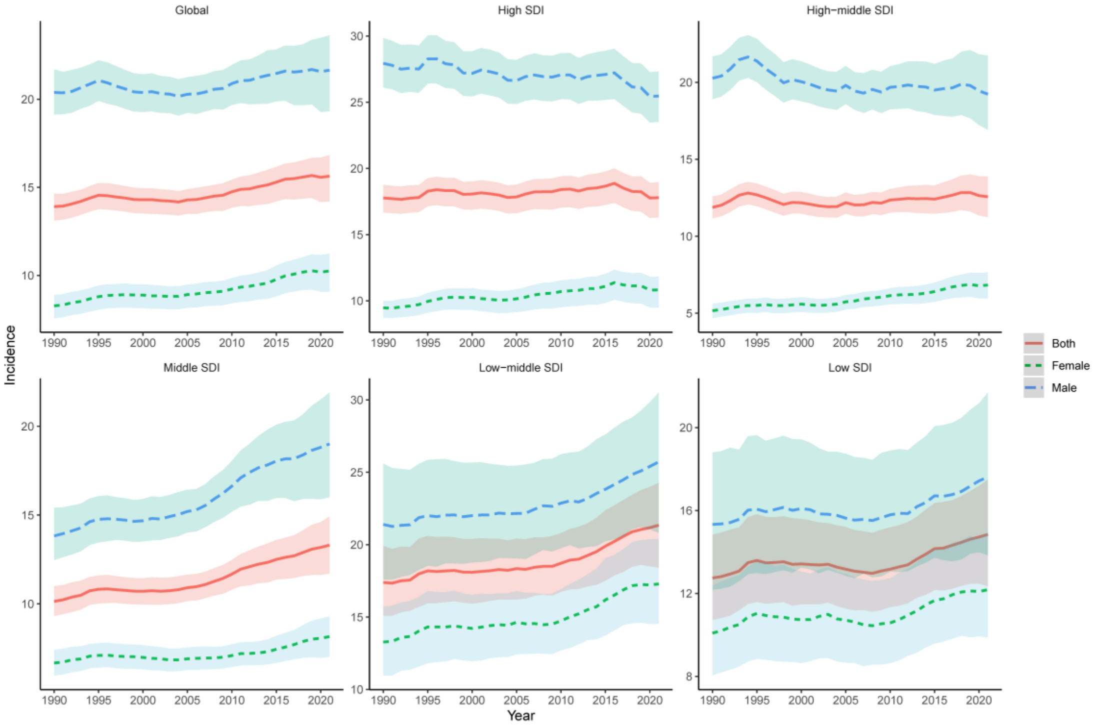

Figure S3: Trends in ASMR by gender for LOCC in Global and Five SDI Regions from 1990 to 2021

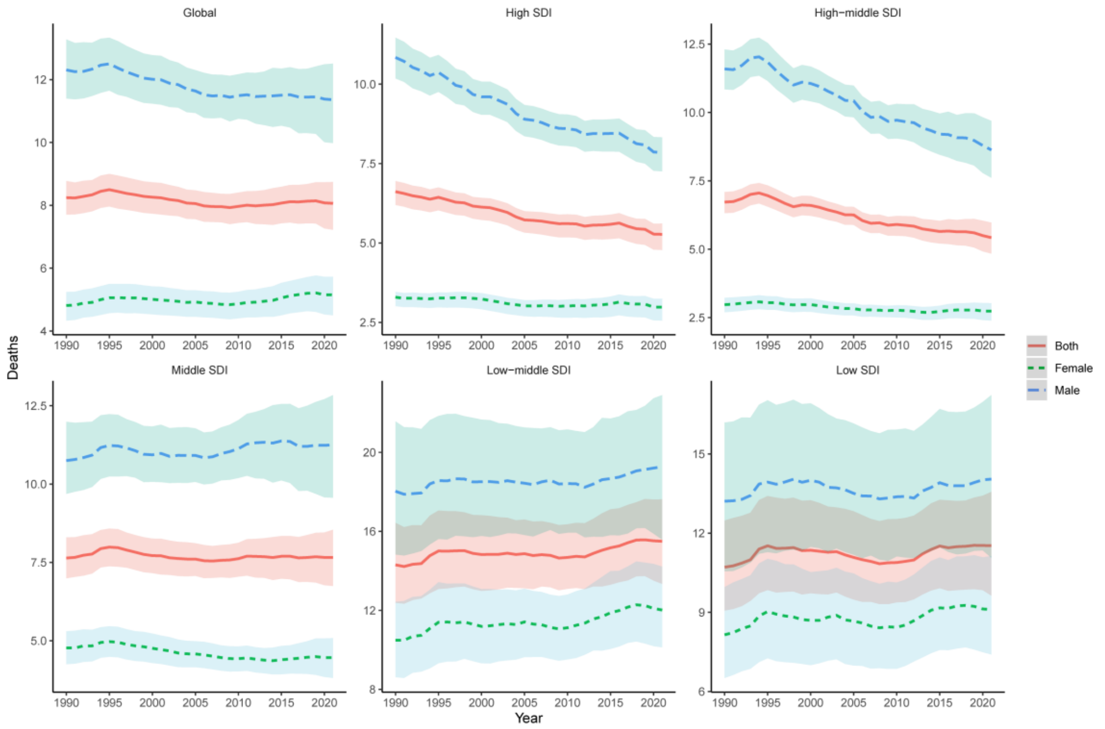

Figure S4: Trends in ASDR by gender for LOCC in Global and Five SDI Regions from 1990 to 2021

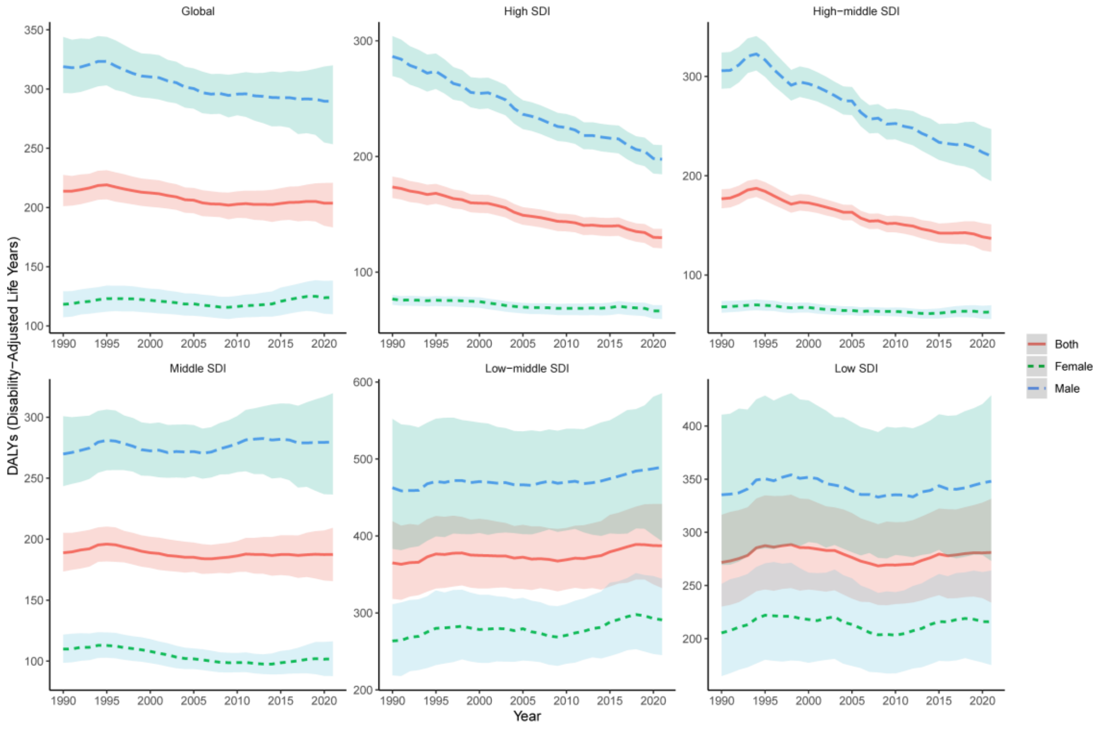

Figure S5: Trends of ASPR in Global and Five SDI Regions at different age group from 1990 to 2021

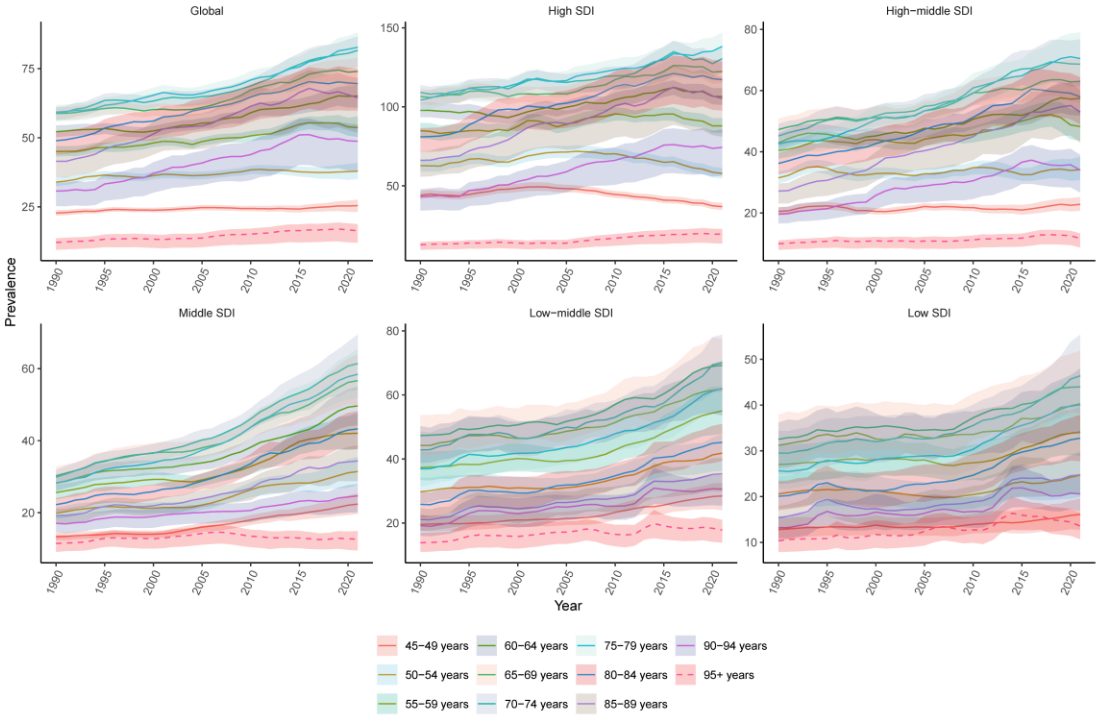

Figure S6: Trends of ASIR in Global and Five SDI Regions at different age group from 1990 to 2021

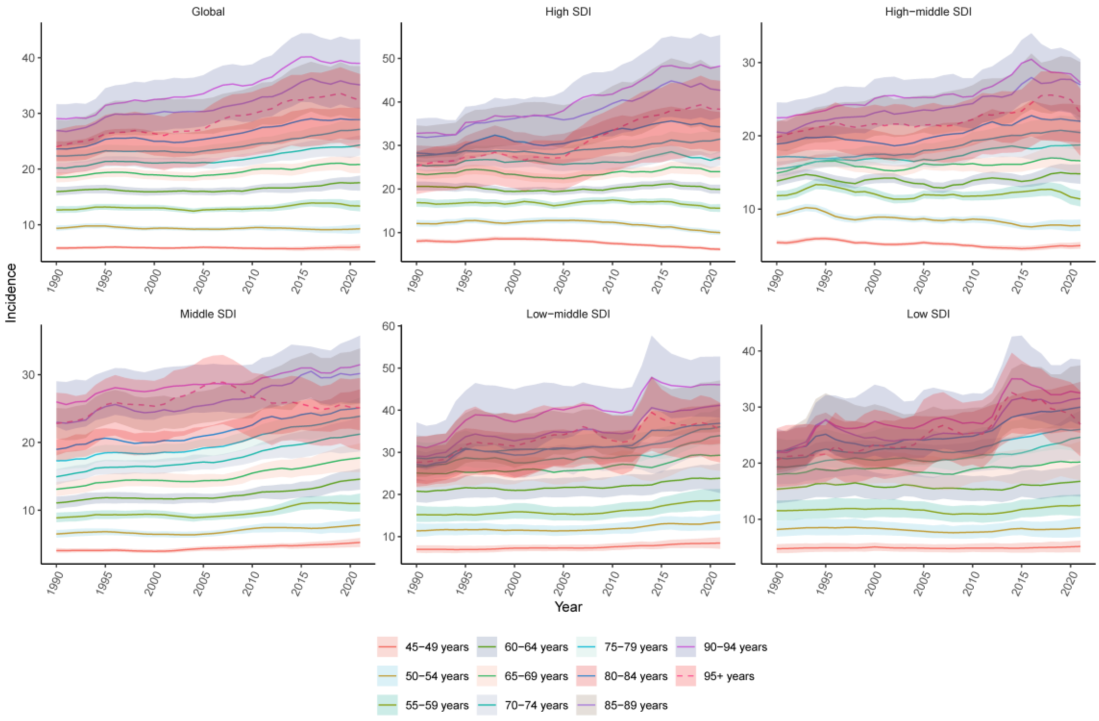

Figure S7: Trends of ASMR in Global and Five SDI Regions at different age group from 1990 to 2021

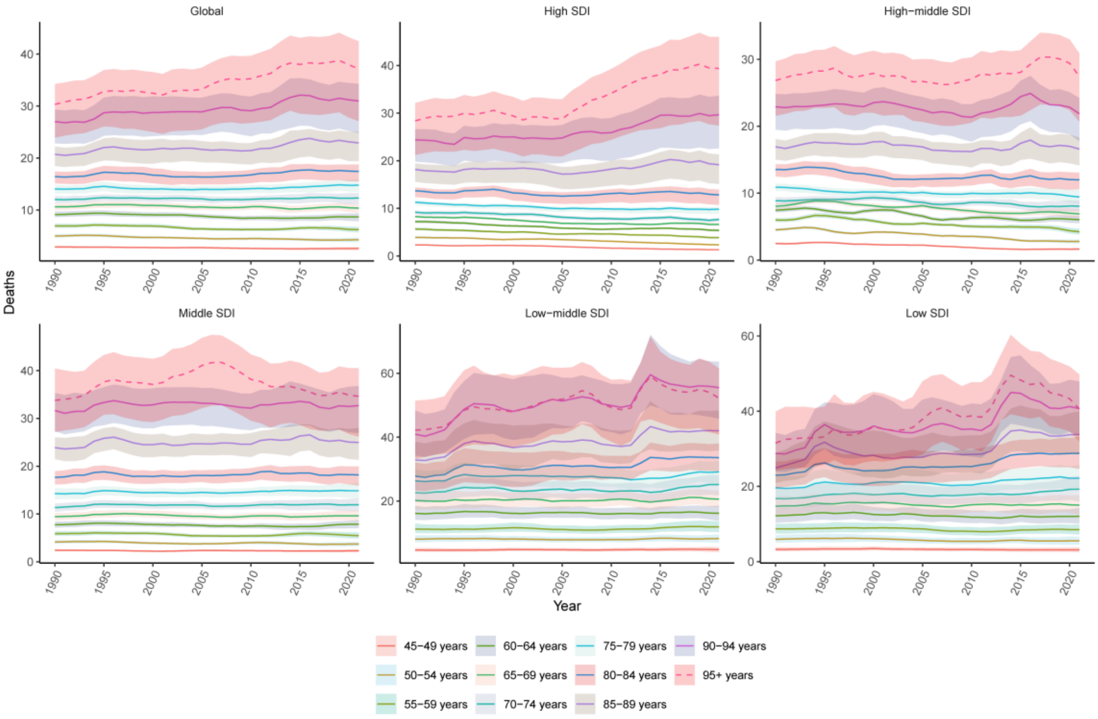

Figure S8: Trends of ASDR in Global and Five SDI Regions at different age group from 1990 to 2021

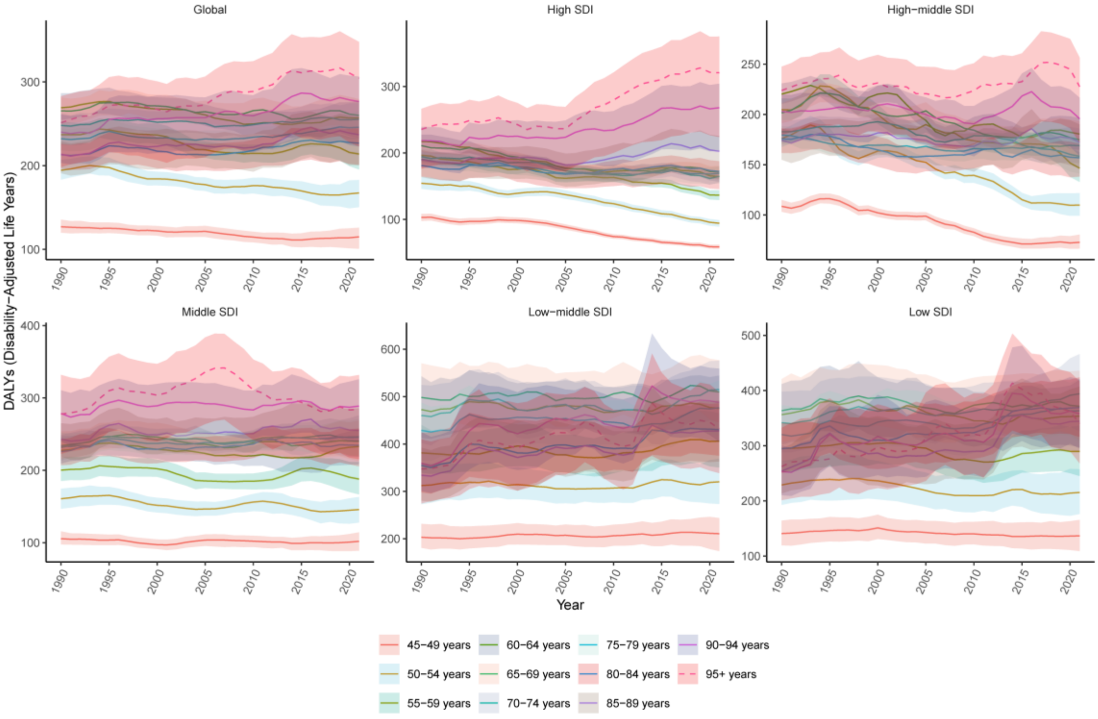

Figure S9: The correlation between EAPC of Age-standardized rates of LOCC and SDI in adults aged 45 and above (A. EAPC of ASPR, B. EAPC of ASIR, C. EAPC of ASMR, D. EAPC of ASDR)

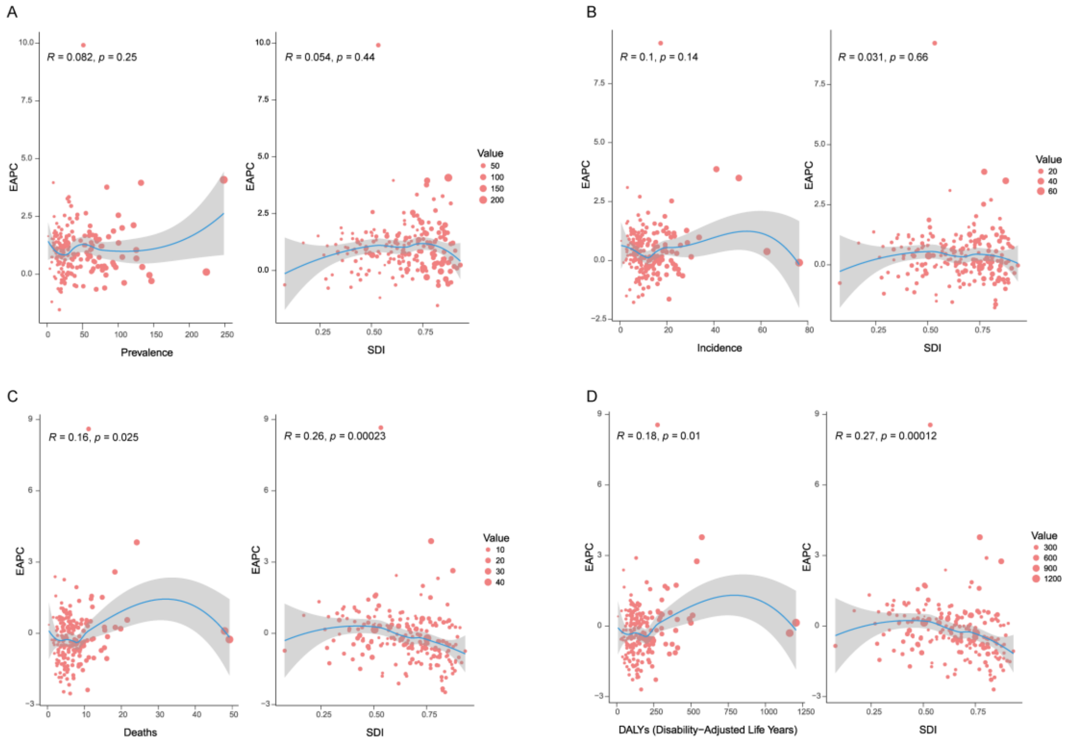

Table S1-S6 captions

Table S1: The EAPC of age-standardized rates of LOCC in different countries

location_name	Prevalence-EAPCs	Incidence-EAPCs	Deaths-EAPCs	DALY-EAPCs
American Samoa	1.78(1.19,2.38)	1.66(1.07,2.24)	1.63(1.03,2.23)	1.66(1.08,2.24)
Antigua and Barbuda	-0.03(-0.34,0.27)	-0.36(-0.67,-0.05)	-0.63(-0.94,-0.33)	-0.82(-1.13,-0.51)
Arab Republic of Egypt	3.97(3.51,4.43)	3.09(2.60,3.59)	2.43(1.92,2.96)	2.39(1.85,2.92)
Argentine Republic	0.44(0.09,0.79)	-0.08(-0.39,0.23)	-0.47(-0.76,-0.18)	-0.74(-1.06,-0.43)
Australia	0.32(0.14,0.50)	-0.21(-0.44,0.01)	-0.97(-1.28,-0.67)	-1.13(-1.42,-0.84)
Barbados	0.31(0.21,0.42)	-0.17(-0.29,-0.06)	-0.47(-0.59,-0.34)	-0.57(-0.68,-0.46)
Belize	0.51(0.20,0.81)	0.24(-0.11,0.60)	-0.04(-0.47,0.38)	0.01(-0.40,0.42)
Bermuda	0.34(0.12,0.56)	-0.79(-1.03,-0.55)	-1.84(-2.14,-1.54)	-1.90(-2.23,-1.58)
Bolivarian Republic of Venezuela	0.81(0.63,0.99)	0.03(-0.12,0.19)	-0.46(-0.64,-0.29)	-0.50(-0.68,-0.32)
Bosnia and Herzegovina	2.42(2.16,2.69)	1.39(1.23,1.56)	0.45(0.34,0.57)	0.44(0.31,0.57)
Brunei Darussalam	0.67(0.52,0.83)	0.12(-0.06,0.30)	-0.34(-0.57,-0.11)	-0.55(-0.77,-0.32)
Burkina Faso	1.09(0.99,1.19)	0.76(0.64,0.88)	0.70(0.57,0.83)	0.61(0.48,0.74)
Canada	-0.59(-0.72,-0.47)	-1.00(-1.14,-0.86)	-1.47(-1.65,-1.29)	-1.66(-1.84,-1.48)
Central African Republic	-0.19(-0.26,-0.11)	-0.32(-0.37,-0.27)	-0.30(-0.36,-0.25)	-0.36(-0.42,-0.30)
Commonwealth of Dominica	0.01(-0.09,0.11)	-0.20(-0.28,-0.12)	-0.30(-0.38,-0.21)	-0.19(-0.27,-0.11)
Commonwealth of the Bahamas	0.29(0.10,0.48)	-0.09(-0.25,0.06)	-0.33(-0.50,-0.17)	-0.52(-0.71,-0.33)
Cook Islands	2.12(1.95,2.28)	1.18(1.05,1.31)	0.34(0.22,0.46)	0.39(0.26,0.52)
Czech Republic	1.67(1.43,1.91)	0.57(0.45,0.69)	-0.33(-0.43,-0.24)	-0.51(-0.62,-0.41)
Democratic People's Republic of Korea	1.08(0.87,1.29)	0.57(0.45,0.68)	0.12(0.05,0.20)	0.15(0.08,0.21)
Democratic Republic of Sao Tome and Principe	1.65(1.54,1.76)	0.83(0.76,0.90)	0.41(0.36,0.46)	0.41(0.33,0.49)
Democratic Republic of the Congo	0.74(0.44,1.04)	0.27(0.06,0.48)	0.12(-0.03,0.28)	0.12(-0.04,0.27)
Democratic Republic of Timor-Leste	1.16(0.90,1.42)	0.57(0.40,0.75)	0.25(0.11,0.38)	0.18(0.02,0.34)
Democratic Socialist Republic of Sri Lanka	2.55(2.26,2.85)	1.27(1.01,1.53)	0.18(-0.06,0.43)	0.57(0.29,0.86)
Dominican Republic	1.34(1.22,1.46)	0.64(0.52,0.76)	0.29(0.15,0.43)	0.38(0.28,0.47)
Eastern Republic of Uruguay	0.65(0.50,0.80)	0.05(-0.07,0.18)	-0.36(-0.47,-0.25)	-0.58(-0.69,-0.46)
Federal Democratic Republic of Ethiopia	0.44(0.28,0.60)	-0.48(-0.60,-0.37)	-0.86(-0.93,-0.78)	-1.04(-1.14,-0.95)
Federal Democratic Republic of Nepal	1.26(0.99,1.53)	0.37(0.13,0.61)	-0.09(-0.31,0.13)	-0.19(-0.41,0.04)

Federal Republic of Germany	0.18(0.03,0.33)	-0.14(-0.26,-0.02)	-1.01(-1.17,-0.86)	-1.47(-1.61,-1.34)
Federal Republic of Nigeria	1.54(1.26,1.82)	1.04(0.86,1.22)	0.71(0.58,0.83)	0.68(0.54,0.83)
Federal Republic of Somalia	-0.63(-0.70,-0.55)	-0.76(-0.83,-0.69)	-0.73(-0.80,-0.66)	-0.84(-0.92,-0.76)
Federated States of Micronesia	1.26(1.14,1.38)	0.84(0.75,0.93)	0.56(0.50,0.62)	0.53(0.46,0.60)
Federative Republic of Brazil	0.88(0.79,0.97)	0.10(-0.00,0.19)	-0.43(-0.53,-0.32)	-0.46(-0.59,-0.34)
French Republic	-0.04(-0.33,0.26)	-0.65(-0.86,-0.45)	-2.48(-2.65,-2.31)	-2.69(-2.93,-2.46)
Gabonese Republic	0.62(0.46,0.79)	0.10(0.02,0.18)	-0.24(-0.34,-0.14)	-0.30(-0.40,-0.20)
Georgia	2.25(1.61,2.90)	2.02(1.41,2.64)	2.00(1.39,2.60)	1.86(1.25,2.48)
Global	0.77(0.72,0.82)	0.35(0.29,0.41)	-0.15(-0.20,-0.09)	-0.25(-0.31,-0.20)
Grand Duchy of Luxembourg	-0.03(-0.23,0.17)	-0.61(-0.74,-0.47)	-1.58(-1.69,-1.46)	-1.88(-2.00,-1.77)
Greenland	-0.65(-0.84,-0.46)	-1.64(-1.80,-1.48)	-2.33(-2.48,-2.19)	-2.28(-2.42,-2.14)
Grenada	0.05(-0.16,0.25)	-0.31(-0.61,-0.02)	-0.61(-0.94,-0.27)	-0.61(-0.86,-0.36)
Guam	3.14(2.84,3.44)	2.70(2.37,3.03)	2.31(1.94,2.68)	2.90(2.57,3.22)
Hashemite Kingdom of Jordan	1.25(1.10,1.40)	0.23(0.12,0.34)	-0.74(-0.85,-0.63)	-0.94(-1.06,-0.81)
Hellenic Republic	1.09(0.99,1.19)	0.85(0.72,0.97)	0.43(0.24,0.61)	0.64(0.45,0.82)
Hungary	1.05(0.58,1.52)	0.15(-0.21,0.52)	-0.67(-0.96,-0.39)	-0.92(-1.32,-0.52)
Independent State of Papua New Guinea	0.95(0.78,1.11)	0.90(0.78,1.01)	0.95(0.86,1.04)	0.89(0.79,0.99)
Independent State of Samoa	0.52(0.38,0.66)	0.05(-0.04,0.14)	-0.18(-0.26,-0.10)	-0.16(-0.25,-0.07)
Ireland	0.35(0.16,0.53)	-0.37(-0.61,-0.13)	-1.70(-1.94,-1.46)	-1.72(-1.95,-1.48)
Islamic Republic of Afghanistan	0.74(0.52,0.96)	0.21(0.08,0.33)	-0.04(-0.10,0.03)	-0.17(-0.23,-0.11)
Islamic Republic of Iran	2.50(2.22,2.78)	1.57(1.37,1.78)	0.73(0.54,0.91)	0.65(0.47,0.82)
Islamic Republic of Mauritania	1.69(1.57,1.80)	0.81(0.71,0.90)	0.34(0.26,0.42)	0.27(0.19,0.36)
Islamic Republic of Pakistan	0.69(0.60,0.77)	0.38(0.26,0.50)	0.15(-0.01,0.32)	0.14(-0.04,0.33)
Jamaica	0.24(-0.11,0.60)	0.01(-0.33,0.35)	-0.14(-0.47,0.18)	-0.33(-0.70,0.05)
Japan	1.66(1.36,1.98)	1.15(0.82,1.48)	0.35(-0.03,0.74)	0.02(-0.39,0.43)
Kingdom of Bahrain	1.39(1.22,1.56)	0.14(-0.01,0.29)	-0.85(-1.03,-0.66)	-1.10(-1.27,-0.94)
Kingdom of Belgium	0.51(0.24,0.78)	0.17(-0.07,0.42)	-0.48(-0.69,-0.28)	-0.75(-0.99,-0.50)
Kingdom of Bhutan	0.94(0.75,1.14)	-0.00(-0.16,0.15)	-0.56(-0.66,-0.45)	-0.77(-0.89,-0.66)
Kingdom of Cambodia	1.69(1.49,1.89)	0.90(0.76,1.03)	0.45(0.36,0.53)	0.33(0.24,0.41)
Kingdom of Denmark	1.21(0.58,1.84)	0.77(0.17,1.38)	-0.15(-0.73,0.44)	-0.39(-0.99,0.21)
Kingdom of Eswatini	0.37(0.30,0.45)	0.45(0.17,0.72)	0.36(0.02,0.70)	0.36(0.00,0.72)

Kingdom of Lesotho	1.28(1.20,1.35)	1.56(1.32,1.81)	1.62(1.33,1.91)	1.69(1.38,2.00)
Kingdom of Morocco	1.70(1.50,1.91)	0.88(0.74,1.03)	0.33(0.24,0.42)	0.30(0.21,0.39)
Kingdom of Norway	0.11(-0.18,0.40)	-0.34(-0.62,-0.06)	-1.24(-1.49,-0.98)	-1.51(-1.77,-1.26)
Kingdom of Saudi Arabia	1.36(1.02,1.69)	0.37(0.24,0.50)	-0.87(-0.97,-0.78)	-0.91(-1.01,-0.81)
Kingdom of Spain	-0.29(-0.39,-0.18)	-0.57(-0.69,-0.46)	-1.47(-1.56,-1.38)	-1.84(-1.93,-1.75)
Kingdom of Sweden	1.18(0.96,1.40)	0.88(0.65,1.11)	0.26(0.00,0.52)	0.04(-0.22,0.30)
Kingdom of Thailand	0.73(0.62,0.84)	-0.25(-0.35,-0.14)	-1.09(-1.21,-0.98)	-1.12(-1.22,-1.01)
Kingdom of the Netherlands	0.53(0.32,0.75)	0.17(-0.00,0.34)	-0.48(-0.61,-0.35)	-0.78(-0.98,-0.58)
Kingdom of Tonga	1.17(1.09,1.24)	0.98(0.93,1.04)	0.86(0.77,0.96)	0.84(0.75,0.92)
Kyrgyz Republic	-0.32(-1.16,0.54)	-0.89(-1.71,-0.07)	-1.32(-2.09,-0.54)	-1.63(-2.39,-0.86)
Lao People's Democratic Republic	0.74(0.58,0.91)	0.04(-0.06,0.15)	-0.32(-0.38,-0.26)	-0.48(-0.54,-0.42)
Lebanese Republic	1.58(1.43,1.73)	0.50(0.36,0.65)	-0.54(-0.68,-0.39)	-0.78(-0.92,-0.65)
Malaysia	0.84(0.56,1.12)	0.12(-0.08,0.32)	-0.47(-0.63,-0.32)	-0.62(-0.85,-0.40)
Mongolia	-0.63(-0.97,-0.29)	-1.55(-1.89,-1.21)	-2.11(-2.45,-1.78)	-2.16(-2.47,-1.85)
Montenegro	1.05(0.81,1.30)	0.78(0.59,0.97)	0.33(0.18,0.48)	0.14(0.01,0.26)
New Zealand	0.41(0.16,0.66)	-0.04(-0.32,0.24)	-0.94(-1.26,-0.61)	-1.05(-1.36,-0.74)
North Macedonia	1.84(1.50,2.19)	0.87(0.59,1.16)	0.09(-0.18,0.37)	-0.07(-0.31,0.17)
Northern Mariana Islands	3.96(3.45,4.47)	3.87(3.35,4.40)	3.88(3.32,4.45)	3.78(3.24,4.32)
Palestine	1.19(1.02,1.35)	0.36(0.29,0.43)	-0.23(-0.35,-0.12)	-0.26(-0.36,-0.17)
People's Democratic Republic of Algeria	1.12(0.99,1.24)	0.24(0.09,0.38)	-0.43(-0.56,-0.30)	-0.62(-0.71,-0.53)
People's Republic of Bangladesh	0.77(0.61,0.92)	-0.36(-0.46,-0.25)	-1.01(-1.09,-0.93)	-1.13(-1.21,-1.06)
People's Republic of China	3.34(3.13,3.56)	1.71(1.48,1.95)	-0.07(-0.23,0.09)	-0.17(-0.34,0.01)
Plurinational State of Bolivia	1.06(1.01,1.12)	0.21(0.15,0.27)	-0.25(-0.29,-0.21)	-0.42(-0.47,-0.37)
Portuguese Republic	2.12(1.89,2.35)	1.03(0.85,1.22)	-0.65(-0.83,-0.48)	-0.46(-0.62,-0.30)
Principality of Andorra	-0.51(-0.73,-0.30)	-0.74(-0.96,-0.52)	-1.31(-1.49,-1.14)	-1.34(-1.51,-1.17)
Principality of Monaco	0.88(0.72,1.03)	0.72(0.58,0.87)	0.24(0.11,0.37)	0.12(-0.00,0.23)
Puerto Rico	-0.47(-0.71,-0.24)	-1.47(-1.69,-1.25)	-2.41(-2.61,-2.21)	-2.28(-2.48,-2.07)
Republic of Albania	2.41(2.12,2.70)	0.98(0.84,1.13)	-0.04(-0.18,0.10)	-0.11(-0.25,0.02)
Republic of Angola	1.34(1.17,1.50)	0.64(0.56,0.72)	0.37(0.33,0.41)	0.28(0.24,0.33)
Republic of Armenia	1.94(1.59,2.28)	1.21(0.93,1.49)	0.63(0.38,0.88)	0.53(0.26,0.81)
Republic of Austria	0.82(0.58,1.06)	0.08(-0.09,0.25)	-0.73(-0.84,-0.62)	-1.15(-1.29,-1.02)
Republic of Azerbaijan	1.30(1.02,1.57)	0.65(0.45,0.84)	0.16(-0.02,0.35)	0.00(-0.16,0.17)
Republic of Belarus	1.22(0.71,1.74)	0.30(-0.09,0.69)	-0.96(-1.24,-0.68)	-0.77(-1.02,-0.51)
Republic of Benin	1.03(0.92,1.14)	0.74(0.63,0.86)	0.62(0.49,0.75)	0.46(0.32,0.59)
Republic of Botswana	0.08(-0.08,0.23)	-0.26(-0.39,-0.13)	-0.49(-0.69,-0.30)	-0.61(-0.83,-0.39)

Republic of Bulgaria	1.62(1.25,2.00)	1.13(0.80,1.47)	0.47(0.14,0.81)	0.58(0.24,0.92)
Republic of Burundi	-0.77(-0.91,-0.62)	-1.20(-1.35,-1.04)	-1.26(-1.41,-1.11)	-1.44(-1.59,-1.28)
Republic of Cabo Verde	9.91(7.34,12.55)	9.22(6.60,11.91)	8.66(5.99,11.40)	8.55(5.89,11.28)
Republic of Cameroon	1.01(0.88,1.14)	0.79(0.70,0.87)	0.66(0.52,0.79)	0.57(0.43,0.71)
Republic of Chad	1.53(1.44,1.61)	1.37(1.23,1.51)	1.33(1.14,1.52)	0.16(0.08,0.25)
Republic of Chile	1.26(1.08,1.45)	0.02(-0.12,0.16)	-1.06(-1.20,-0.92)	1.32(1.12,1.52)
Republic of Colombia	0.41(0.18,0.64)	-0.93(-1.17,-0.69)	-1.80(-2.02,-1.58)	-1.23(-1.35,-1.11)
Republic of Costa Rica	-0.37(-0.58,-0.16)	-0.82(-1.01,-0.64)	-1.25(-1.40,-1.09)	-1.85(-2.09,-1.61)
Republic of Cote d'Ivoire	0.81(0.65,0.97)	0.41(0.33,0.48)	0.17(0.10,0.24)	-1.46(-1.64,-1.27)
Republic of Croatia	0.75(0.64,0.85)	-0.14(-0.24,-0.04)	-1.34(-1.45,-1.24)	-1.54(-1.64,-1.45)
Republic of Cuba	1.43(1.26,1.60)	0.67(0.51,0.83)	0.16(-0.00,0.32)	0.36(0.19,0.53)
Republic of Cyprus	1.46(1.25,1.67)	0.16(0.02,0.31)	-1.61(-1.78,-1.44)	-1.14(-1.23,-1.04)
Republic of Djibouti	0.00(-0.13,0.14)	-0.23(-0.31,-0.16)	-0.37(-0.46,-0.27)	-0.53(-0.63,-0.42)
Republic of Ecuador	2.20(2.01,2.40)	1.20(1.01,1.38)	0.62(0.41,0.83)	0.45(0.24,0.66)
Republic of El Salvador	1.76(1.67,1.86)	0.39(0.29,0.50)	-0.44(-0.58,-0.30)	-0.46(-0.62,-0.31)
Republic of Equatorial Guinea	2.55(2.40,2.69)	1.09(1.03,1.15)	0.28(0.24,0.32)	0.09(0.02,0.15)
Republic of Estonia	1.87(1.57,2.17)	0.99(0.69,1.29)	-0.41(-0.75,-0.07)	-0.64(-1.01,-0.27)
Republic of Fiji	0.09(-0.11,0.30)	0.05(-0.07,0.17)	0.04(-0.04,0.12)	-0.02(-0.12,0.09)
Republic of Finland	1.37(1.19,1.56)	0.70(0.55,0.84)	-0.48(-0.60,-0.36)	-0.55(-0.64,-0.47)
Republic of Ghana	0.24(-0.14,0.63)	-0.09(-0.39,0.21)	-0.22(-0.48,0.03)	-0.56(-0.86,-0.26)
Republic of Guatemala	-0.10(-0.30,0.09)	-1.24(-1.44,-1.04)	-1.80(-2.00,-1.61)	-1.73(-1.93,-1.52)
Republic of Guinea	0.82(0.77,0.88)	0.64(0.61,0.68)	0.56(0.51,0.61)	0.54(0.49,0.59)
Republic of Guinea-Bissau	1.38(1.30,1.47)	1.12(1.01,1.23)	1.03(0.89,1.17)	0.88(0.74,1.02)
Republic of Guyana	0.57(0.40,0.75)	0.11(-0.05,0.28)	-0.08(-0.23,0.07)	0.04(-0.13,0.21)
Republic of Haiti	0.09(-0.03,0.21)	-0.20(-0.24,-0.15)	-0.31(-0.35,-0.26)	-0.40(-0.45,-0.35)
Republic of Honduras	1.80(1.71,1.90)	1.30(1.19,1.42)	1.00(0.87,1.13)	0.92(0.81,1.03)
Republic of Iceland	0.36(0.26,0.45)	0.09(-0.02,0.19)	-0.56(-0.67,-0.45)	-0.85(-0.96,-0.74)
Republic of India	1.51(1.30,1.73)	0.75(0.59,0.91)	0.25(0.13,0.36)	0.14(0.03,0.24)
Republic of Indonesia	0.94(0.79,1.08)	0.61(0.57,0.66)	0.39(0.33,0.45)	0.19(0.14,0.24)
Republic of Iraq	1.32(1.04,1.59)	0.48(0.32,0.64)	-0.30(-0.40,-0.20)	-0.52(-0.60,-0.44)
Republic of Italy	0.15(0.01,0.28)	-0.57(-0.68,-0.46)	-1.36(-1.55,-1.16)	-1.69(-1.87,-1.51)
Republic of Kazakhstan	0.02(-0.42,0.46)	-0.67(-0.98,-0.36)	-1.29(-1.54,-1.04)	-1.54(-1.79,-1.29)
Republic of Kenya	1.50(1.32,1.68)	1.18(1.08,1.29)	1.16(1.03,1.30)	1.11(0.97,1.26)
Republic of Kiribati	0.62(0.58,0.67)	0.39(0.36,0.42)	0.31(0.28,0.35)	0.28(0.25,0.31)
Republic of Korea	2.96(2.43,3.48)	1.08(0.72,1.44)	-1.23(-1.56,-0.89)	-1.37(-1.65,-1.10)
Republic of Latvia	2.09(1.79,2.40)	1.25(1.02,1.49)	0.46(0.26,0.66)	0.27(0.05,0.48)
Republic of Liberia	1.88(1.77,1.99)	1.10(1.00,1.19)	0.73(0.64,0.83)	0.68(0.58,0.79)
Republic of Lithuania	1.70(1.41,1.99)	1.16(0.90,1.41)	0.54(0.31,0.76)	0.49(0.23,0.74)
Republic of Madagascar	-0.73(-0.93,-0.53)	-1.01(-1.17,-0.85)	-1.10(-1.23,-0.96)	-1.19(-1.33,-1.06)
Republic of Malawi	1.00(0.89,1.10)	0.40(0.32,0.47)	0.19(0.10,0.28)	0.17(0.07,0.27)

Republic of Maldives	0.73(0.47,0.99)	-0.66(-0.81,-0.50)	-1.71(-1.86,-1.56)	-2.05(-2.21,-1.89)
Republic of Mali	0.69(0.54,0.83)	0.31(0.19,0.44)	0.16(0.05,0.28)	0.10(-0.02,0.22)
Republic of Malta	0.57(0.46,0.68)	0.00(-0.10,0.11)	-1.00(-1.10,-0.89)	-0.92(-1.03,-0.82)
Republic of Mauritius	0.64(0.29,1.00)	0.22(-0.13,0.56)	-0.08(-0.42,0.27)	0.00(-0.33,0.33)
Republic of Moldova	0.85(0.43,1.27)	0.19(-0.14,0.52)	-0.62(-0.87,-0.37)	-0.64(-0.89,-0.39)
Republic of Mozambique	1.15(1.05,1.25)	0.93(0.86,1.01)	0.84(0.74,0.93)	0.92(0.81,1.02)
Republic of Namibia	1.25(1.15,1.34)	0.64(0.52,0.77)	0.30(0.13,0.48)	0.30(0.12,0.49)
Republic of Nauru	0.98(0.73,1.23)	0.73(0.65,0.81)	0.58(0.50,0.66)	0.54(0.46,0.62)
Republic of Nicaragua	1.34(1.22,1.47)	0.43(0.29,0.58)	-0.19(-0.35,-0.02)	-0.16(-0.31,-0.02)
Republic of Niue	1.19(1.07,1.31)	0.83(0.78,0.88)	0.55(0.50,0.60)	0.49(0.45,0.54)
Republic of Palau	0.10(0.01,0.19)	-0.09(-0.15,-0.04)	-0.21(-0.26,-0.15)	-0.30(-0.33,-0.26)
Republic of Panama	0.49(0.32,0.67)	-0.30(-0.44,-0.16)	-0.79(-0.89,-0.68)	-0.84(-0.95,-0.72)
Republic of Paraguay	1.64(1.53,1.76)	1.25(1.12,1.39)	0.89(0.73,1.05)	0.87(0.69,1.05)
Republic of Peru	2.04(1.78,2.30)	0.48(0.21,0.75)	-0.63(-0.86,-0.40)	-0.70(-0.94,-0.45)
Republic of Poland	2.64(2.50,2.79)	1.52(1.43,1.61)	0.60(0.49,0.72)	0.58(0.49,0.68)
Republic of Rwanda	-0.17(-0.44,0.10)	-1.14(-1.37,-0.91)	-1.50(-1.72,-1.27)	-1.71(-1.96,-1.47)
Republic of San Marino	-0.52(-0.88,-0.15)	-0.74(-1.10,-0.39)	-1.15(-1.50,-0.80)	-1.14(-1.47,-0.82)
Republic of Senegal	1.64(1.54,1.73)	1.25(1.15,1.34)	1.10(1.01,1.20)	1.00(0.90,1.10)
Republic of Serbia	1.84(1.70,1.98)	0.58(0.49,0.67)	-0.61(-0.71,-0.51)	-0.46(-0.59,-0.34)
Republic of Seychelles	1.36(1.03,1.68)	0.98(0.67,1.29)	0.62(0.31,0.92)	0.46(0.16,0.75)
Republic of Sierra Leone	1.65(1.60,1.69)	1.25(1.19,1.32)	1.11(1.00,1.22)	1.09(0.98,1.19)
Republic of Singapore	1.60(1.27,1.92)	0.41(0.11,0.71)	-1.11(-1.41,-0.82)	-1.38(-1.65,-1.10)
Republic of Slovenia	0.28(0.12,0.43)	-0.54(-0.66,-0.42)	-1.87(-1.98,-1.75)	-2.18(-2.30,-2.06)
Republic of South Africa	0.03(-0.08,0.14)	-0.23(-0.37,-0.08)	-0.51(-0.75,-0.27)	-0.56(-0.80,-0.32)
Republic of South Sudan	-0.37(-0.57,-0.17)	-0.53(-0.67,-0.39)	-0.62(-0.73,-0.50)	-0.75(-0.89,-0.61)
Republic of Sudan	0.74(0.52,0.97)	0.00(-0.12,0.12)	-0.43(-0.50,-0.36)	-0.59(-0.66,-0.52)
Republic of Suriname	0.41(0.25,0.57)	-0.06(-0.21,0.09)	-0.29(-0.42,-0.15)	-0.26(-0.39,-0.12)
Republic of Tajikistan	-1.19(-1.33,-1.05)	-1.51(-1.69,-1.33)	-1.68(-1.91,-1.44)	-1.57(-1.76,-1.38)
Republic of the Congo	0.72(0.54,0.91)	0.10(-0.03,0.23)	-0.19(-0.29,-0.09)	-0.27(-0.38,-0.16)
Republic of the Gambia	0.91(0.69,1.13)	0.67(0.51,0.82)	0.49(0.36,0.62)	0.44(0.28,0.61)
Republic of the Marshall Islands	1.14(1.04,1.25)	1.03(0.94,1.13)	0.95(0.84,1.05)	0.91(0.80,1.01)
Republic of the Niger	1.22(1.12,1.32)	0.91(0.82,1.00)	0.75(0.65,0.86)	0.65(0.53,0.77)
Republic of the Philippines	-0.24(-0.36,-0.12)	-0.40(-0.47,-0.33)	-0.48(-0.56,-0.39)	-0.51(-0.60,-0.42)
Republic of the Union of Myanmar	0.83(0.62,1.05)	-0.08(-0.18,0.02)	-0.51(-0.56,-0.46)	-0.66(-0.72,-0.61)
Republic of Trinidad and Tobago	-0.02(-0.21,0.17)	-0.80(-0.98,-0.62)	-1.33(-1.50,-1.16)	-1.22(-1.40,-1.04)
Republic of Tunisia	1.19(1.08,1.31)	0.37(0.32,0.42)	-0.44(-0.50,-0.37)	-0.46(-0.52,-0.40)
Republic of Turkey	1.53(1.40,1.66)	0.03(-0.11,0.17)	-1.20(-1.39,-1.02)	-1.41(-1.57,-1.26)

Republic of Uganda	0.42(0.29,0.55)	-0.03(-0.17,0.12)	-0.21(-0.38,-0.04)	-0.34(-0.53,-0.15)
Republic of Uzbekistan	0.79(0.44,1.13)	0.60(0.35,0.84)	0.29(0.08,0.50)	0.29(0.07,0.50)
Republic of Vanuatu	0.76(0.57,0.95)	0.71(0.61,0.80)	0.70(0.63,0.77)	0.67(0.58,0.76)
Republic of Yemen	0.52(0.39,0.65)	0.06(-0.00,0.13)	-0.22(-0.27,-0.16)	-0.46(-0.52,-0.40)
Republic of Zambia	2.57(1.97,3.19)	1.87(1.45,2.29)	1.57(1.23,1.91)	1.74(1.35,2.13)
Republic of Zimbabwe	1.06(0.78,1.33)	1.20(0.79,1.60)	1.42(1.01,1.82)	1.57(1.12,2.01)
Romania	3.77(3.46,4.08)	2.52(2.27,2.78)	1.51(1.27,1.74)	1.60(1.32,1.89)
Russian Federation	1.26(0.90,1.62)	0.35(0.05,0.66)	-0.71(-1.01,-0.41)	-0.77(-1.11,-0.43)
Saint Kitts and Nevis	1.07(0.84,1.31)	0.14(-0.08,0.37)	-0.43(-0.66,-0.19)	-0.50(-0.78,-0.22)
Saint Lucia	-0.07(-0.25,0.11)	-0.79(-1.04,-0.55)	-1.25(-1.55,-0.96)	-1.10(-1.37,-0.84)
Saint Vincent and the Grenadines	0.33(0.09,0.58)	0.03(-0.24,0.30)	-0.17(-0.43,0.09)	-0.09(-0.34,0.16)
Slovak Republic	0.30(0.24,0.36)	-0.38(-0.44,-0.32)	-0.99(-1.07,-0.91)	-1.18(-1.29,-1.08)
Socialist Republic of Viet Nam	1.54(1.36,1.72)	0.55(0.39,0.71)	-0.25(-0.40,-0.10)	-0.18(-0.30,-0.05)
Solomon Islands	1.38(1.15,1.61)	1.06(0.90,1.21)	0.91(0.79,1.02)	0.98(0.84,1.12)
State of Eritrea	-0.07(-0.21,0.06)	-0.38(-0.50,-0.26)	-0.46(-0.59,-0.34)	-0.66(-0.78,-0.54)
State of Israel	0.87(0.64,1.11)	0.25(0.03,0.47)	-0.75(-0.97,-0.53)	-0.81(-1.02,-0.61)
State of Kuwait	0.54(-0.77,1.86)	-0.15(-1.38,1.10)	-0.79(-1.97,0.39)	-1.31(-2.50,-0.10)
State of Libya	1.18(1.03,1.33)	0.72(0.58,0.87)	0.14(0.04,0.24)	0.07(-0.02,0.17)
State of Qatar	3.27(2.78,3.77)	1.92(1.33,2.52)	0.48(-0.21,1.17)	0.46(-0.17,1.09)
Sultanate of Oman	1.18(0.94,1.42)	0.23(0.07,0.38)	-0.66(-0.83,-0.48)	-0.82(-1.00,-0.64)
Swiss Confederation	0.24(-0.20,0.69)	-0.02(-0.42,0.38)	-0.75(-1.03,-0.47)	-1.07(-1.39,-0.74)
Syrian Arab Republic	1.37(1.21,1.52)	0.48(0.38,0.58)	-0.34(-0.45,-0.24)	-0.52(-0.62,-0.42)
Taiwan (Province of China)	4.09(3.48,4.70)	3.50(2.93,4.07)	2.64(2.08,3.19)	2.75(2.14,3.37)
Togolese Republic	1.41(1.30,1.51)	1.17(1.09,1.25)	1.06(0.94,1.18)	0.95(0.83,1.07)
Tokelau	1.46(1.34,1.59)	0.80(0.75,0.84)	0.34(0.28,0.39)	0.36(0.31,0.42)
Turkmenistan	0.34(-0.14,0.82)	-0.26(-0.66,0.15)	-0.64(-1.01,-0.27)	-0.65(-1.02,-0.29)
Tuvalu	1.62(1.58,1.66)	1.02(0.99,1.06)	0.71(0.66,0.77)	0.72(0.67,0.77)
Ukraine	1.67(1.42,1.93)	1.17(0.93,1.42)	0.62(0.38,0.87)	0.64(0.37,0.91)
Union of the Comoros	-0.17(-0.31,-0.04)	-0.47(-0.58,-0.35)	-0.63(-0.75,-0.51)	-0.81(-0.96,-0.67)
United Arab Emirates	1.48(1.04,1.93)	1.52(1.05,2.00)	1.21(0.71,1.72)	0.30(-0.11,0.72)
United Kingdom of Great Britain and Northern Ireland	2.00(1.88,2.13)	1.38(1.25,1.52)	0.25(0.07,0.43)	0.22(0.06,0.37)
United Mexican States	0.68(0.55,0.81)	-0.29(-0.40,-0.17)	-0.87(-0.97,-0.78)	-0.75(-0.85,-0.65)
United Republic of Tanzania	-0.41(-0.54,-0.28)	-0.76(-0.85,-0.68)	-0.90(-0.97,-0.82)	-0.99(-1.08,-0.91)
United States of America	-0.37(-0.45,-0.29)	-0.61(-0.71,-0.50)	-1.19(-1.37,-1.01)	-1.41(-1.59,-1.23)
United States Virgin Islands	-1.53(-1.78,-1.29)	-1.78(-2.05,-1.51)	-1.99(-2.27,-1.71)	-2.07(-2.34,-1.81)

Table S2: The APC and AAPC results of age-standardized rates of LOCC

	Prevalence		
	row.names	APCs	p
slope1	slope1	0.895(0.707,1.084)	*
slope2	slope2	0.371(0.270,0.472)	*
slope3	slope3	1.118(1.052,1.184)	*
slope4	slope4	0.309(0.061,0.557)	*
"AAPC= 0.355(0.342,0.368)"			
	Incidence		
	row.names	APCs	p
slope1	slope1	0.951(0.810,1.092)	*
slope2	slope2	-0.280(-0.355,-0.205)	*
slope3	slope3	0.729(0.685,0.772)	*
slope4	slope4	0.033(-0.227,0.294)	
"AAPC= 0.057(0.054,0.060)"			
	Death		
	row.names	APCs	p
slope1	slope1	0.650(0.482,0.819)	*
slope2	slope2	-0.510(-0.545,-0.475)	*
slope3	slope3	0.280(0.221,0.338)	*
slope4	slope4	-0.498(-0.871,-0.124)	*
"AAPC= -0.005(-0.006,-0.003)"			
	DALYs		
	row.names	APCs	p
slope1	slope1	0.561(0.390,0.733)	*
slope2	slope2	-0.598(-0.633,-0.562)	*
slope3	slope3	0.142(0.083,0.202)	*
slope4	slope4	-0.368(-0.747,0.013)	
"AAPC= -0.315(-0.358,-0.271)"			

APC,annual percentage change ;AAPC,average annual percentage change.

Table S3: The correlation coefficient and its p value between LOCC burden indicators and SDI

21 GBD regions SDI: "r=0.6945, p=0.000e+00"<br>204 countries SDI: "r=0.6760, p=0.000e+00"<br><br>ASIR<br>21 GBD regions SDI: "r=0.5316, p=0.000e+00"<br>204 countries SDI: "r=0.4319, p=1.620e-10"<br><br>ASMR<br>21 GBD regions SDI: "r=-0.0358, p=3.433e-01"<br>204 countries SDI: "r=-0.0736, p=2.955e-01"<br><br>ASDR<br>21 GBD regions SDI: "r=-0.0143, p=7.052e-01"<br>204 countries SDI: "r=-0.0713, p=3.106e-01"

ASIR,age-standardized incidence rate; ASPR, age-standardized prevalence rate; ASMR, age-standardized mortality rate;ASDR,age-standardized DALY rate

Table S4: Decomposition Analysis of Disease Burden across five SDI and 21 GBD regions

location_name	group	Overall_Difference	Aging	Population	Epidemiological_Change
Southeast Asia	ASPR	61028.99	9543.2	33081.28	18404.51
East Asia	ASPR	184139.33	18601.48	75996.53	89541.32
Oceania	ASPR	263.74	45.86	166.92	50.95
Global	ASPR	815096.31	9168.2	620022.34	185905.77
Central Asia	ASPR	2594.4	-680.87	3089.57	185.7
Central Europe	ASPR	23004.57	-17483.23	21693.6	18794.19
Eastern Europe	ASPR	24915.86	-26745.38	31217.89	20443.34
Australasia	ASPR	10218.37	91.76	8901.71	1224.9
Western Europe	ASPR	87707.44	-67378.83	129744.34	25341.93
High-income Asia Pacific	ASPR	36804.14	-7449.04	24964.26	19288.91
High-income North America	ASPR	74142.77	-38121.63	113661.77	-1397.37
Southern Latin America	ASPR	3299.17	-1539.78	3902.02	936.92
Caribbean	ASPR	3763.27	-644.47	3307.63	1100.11
Andean Latin America	ASPR	1970.6	383.28	931.6	655.72

Tropical Latin America	ASPR	19885.66	2252.31	11625.22	6008.13
Central Latin America	ASPR	8892.26	1863.91	5088.13	1940.22
North Africa and Middle East	ASPR	14128.13	2386.76	6839.12	4902.25
Eastern Sub-Saharan Africa	ASPR	7744.65	496.31	5723	1525.34
South Asia	ASPR	241416.31	28442.26	136105.34	76868.71
Southern Sub-Saharan Africa	ASPR	3403.87	88.64	2833.71	481.52
Western Sub-Saharan Africa	ASPR	3945.64	294.03	2441.13	1210.48
Central Sub-Saharan Africa	ASPR	1827.14	246.3	1165.65	415.19
Low SDI	ASPR	31076.78	1341.64	21641.21	8093.93
High SDI	ASPR	226108.18	-67259.14	254878.2	38489.13
Middle SDI	ASPR	251430.6	33975.17	123720.86	93734.57
Low-middle SDI	ASPR	148605.25	10159.28	90752.53	47693.44
High-middle SDI	ASPR	157236.49	-15570.42	126932.64	45874.27
Global	ASIR	222114.75	7688.64	186165.9	28260.21
Southeast Asia	ASIR	16692.75	3304.63	10944.54	2443.58
East Asia	ASIR	44668.86	6980.83	22917.48	14770.55
Central Europe	ASIR	5135.3	-4441.47	6560.1	3016.67
Oceania	ASIR	96.4	17.66	64.43	14.3
Central Asia	ASIR	706.29	-275.13	1113.87	-132.45
High-income Asia Pacific	ASIR	11964.76	724.05	7463.22	3777.49
Western Europe	ASIR	14867.06	-13085.03	28264.24	-312.15
High-income North America	ASIR	12534.26	-3928.22	21911.62	-5449.14
Australasia	ASIR	1698.06	-34.29	1770.67	-38.32
Eastern Europe	ASIR	7075.61	-8917.03	11895.55	4097.09
Southern Latin America	ASIR	765.04	-239.45	1239.05	-234.56
Caribbean	ASIR	1020.52	-18.68	1087.19	-48
High SDI	ASIR	46069.29	-12042.81	56407.46	1704.65
Andean Latin America	ASIR	584.97	148.77	398.35	37.85
Eastern Sub-Saharan Africa	ASIR	2956.93	212.25	2635.17	109.51
Central Latin America	ASIR	2636.29	936.76	1809.72	-110.19

Tropical Latin America	ASIR	5935.36	1561.6	4089.71	284.05
North Africa and Middle East	ASIR	3653.8	579.42	2513.29	561.1
Central Sub-Saharan Africa	ASIR	709.2	106.32	528.53	74.35
South Asia	ASIR	85623.82	14224.75	56647.86	14751.21
Western Sub-Saharan Africa	ASIR	1557.49	99.26	1096.13	362.11
Low SDI	ASIR	12354.64	672.63	9948.68	1733.32
High-middle SDI	ASIR	36497.41	-3424.54	37221.32	2700.63
Middle SDI	ASIR	71812.75	13423.03	42633.28	15756.44
Low-middle SDI	ASIR	55231.51	5490.02	39269.86	10471.63
Southern Sub-Saharan Africa	ASIR	1231.97	21.59	1128.41	81.96
High-income Asia Pacific	ASMR	3960.02	798.46	2526.34	635.22
Central Asia	ASMR	368.75	-201.8	775.75	-205.21
Global	ASMR	103259.41	6131.74	101270.5	-4142.83
East Asia	ASMR	15942	4210.98	12121.82	-390.8
Australasia	ASMR	300.44	52.29	418.8	-170.65
Central Sub-Saharan Africa	ASMR	518.22	83.72	416.3	18.21
Western Sub-Saharan Africa	ASMR	1090.86	65.19	835.91	189.76
Low SDI	ASMR	8608.69	554.65	7646.93	407.11
Tropical Latin America	ASMR	3297.97	1119.24	2665.31	-486.58
Oceania	ASMR	67.08	12.73	45.81	8.55
Low-middle SDI	ASMR	36150.77	4524.06	29097.18	2529.52
Andean Latin America	ASMR	314.55	110.86	239.42	-35.73
High-income North America	ASMR	2129.98	-975.73	6027.45	-2921.74
High-middle SDI	ASMR	11681.93	-1086.16	18227.86	-5459.76
High SDI	ASMR	11231.07	-2904.19	19354.91	-5219.65
Western Europe	ASMR	2019.31	-3987.19	10367.51	-4361.01
North Africa and Middle East	ASMR	1691.43	480.74	1344.79	-134.11
Eastern Europe	ASMR	1786.3	-4672.37	6142.87	315.8
Central Europe	ASMR	2006.09	-2136.84	3772.71	370.22
Middle SDI	ASMR	35524.27	9106.47	26603.95	-186.15
Southeast Asia	ASMR	8845.84	2185.37	6979.44	-318.97
Caribbean	ASMR	530.45	8.94	668.73	-147.22

Central Latin America	ASMR	1439.49	664.05	1209.12	-433.69
Eastern Sub-Saharan Africa	ASMR	2098.63	167.21	2067.66	-136.23
Southern Sub-Saharan Africa	ASMR	777.09	8.05	782.25	-13.2
Southern Latin America	ASMR	286.53	-104.89	696.97	-305.55
South Asia	ASMR	53788.37	11197.36	40906.26	1684.75
Global	ASDR	2517594.38	28033.23	2684524.98	-194963.82
Tropical Latin America	ASDR	82856.53	24080.76	71214.72	-12438.96
South Asia	ASDR	1415895.7	114849.73	1161914.8	139131.18
High-income North America	ASDR	36820.35	-24751.96	143538.16	-81965.85
Central Latin America	ASDR	32631.19	13649.71	28107.75	-9126.27
East Asia	ASDR	377138.4	79067.64	313772.38	-15701.62
Central Europe	ASDR	42495.84	-68896.94	101501.2	9891.57
North Africa and Middle East	ASDR	42770.22	12038.77	34878.81	-4147.35
High-income Asia Pacific	ASDR	55565.21	-1276.63	52228.9	4612.94
Eastern Sub-Saharan Africa	ASDR	58532.05	5057.27	57967.81	-4493.02
Middle SDI	ASDR	893893.09	195747.77	711114.79	-12969.47
Caribbean	ASDR	12858.27	-448.98	16068.24	-2760.99
Southeast Asia	ASDR	219860.43	51667.18	179430.76	-11237.51
High-middle SDI	ASDR	258029.49	-58130.7	483306.02	-167145.83
Western Sub-Saharan Africa	ASDR	31019.98	2750.08	23308	4961.89
High SDI	ASDR	187141.93	-114416.17	467148.7	-165590.6
Oceania	ASDR	1945.08	367.96	1328.25	248.87
Low-middle SDI	ASDR	946910.19	91579.86	799976.5	55353.83
Eastern Europe	ASDR	42067.89	-142633.74	174015.87	10685.77
Southern Sub-Saharan Africa	ASDR	22362.33	628.53	22314	-580.2
Central Asia	ASDR	9927.47	-4601.23	21302.35	-6773.65
Central Sub-Saharan Africa	ASDR	15038.77	2545.91	12003.56	489.3
Low SDI	ASDR	230222.41	13628.07	212884.69	3709.65
Western Europe	ASDR	480.03	-121012.66	254182.54	-132689.86
Australasia	ASDR	5583.89	310.44	9930.92	-4657.47

Andean Latin America	ASDR	7005.32	2331.78	5679.22	-1005.69
Southern Latin America	ASDR	4739.44	-3824.7	17456.06	-8891.93

Table S5: BAPC results of LOCC disease burden among adults aged 45 and older

Time	val-ASPR	val-ASIR	val-ASMR	val-ASDR
1990	42.84771278511811	13.92367858307665	8.259723498208654	213.8458884878463
1991	43.07873749471756	13.96968573137157	8.265382753389071	213.8789275498942
1992	43.46653722959663	14.09226630208967	8.312405262798025	215.0591197461867
1993	43.94535598426766	14.2415314537004	8.36950519285269	216.5127180237871
1994	44.36277157688221	14.41402070876026	8.46415848186683	218.59567359381
1995	44.92639986493439	14.55848437614928	8.503371584589873	219.1142495662726
1996	45.02807639297041	14.53977954129762	8.4570239616241	217.3363783155065
1997	44.97142509311856	14.47388407024173	8.403813783166283	215.6803969859965
1998	44.96145579894065	14.41483498915274	8.361993760921699	214.2503786407918
1999	44.96034721980513	14.33862760834952	8.31533851697793	212.9728570730723
2000	45.46404380021137	14.32558590748592	8.289231878482386	212.3800206087842
2001	45.99028191173851	14.32673349417192	8.266239953340131	211.6740985764371
2002	46.10868823511571	14.2781215105842	8.21507511961292	210.141688683262
2003	46.14001794394697	14.25381483183569	8.177758024869531	208.9988975062328
2004	46.1353984087599	14.20855498522443	8.105629179520598	206.6436575120256
2005	46.66491713326391	14.31746171751502	8.083813515172128	206.2286096636961
2006	47.05819786579482	14.34476301534107	8.014453795838625	203.9875678607609
2007	47.63186913565292	14.43237577605654	7.988977241087571	203.0912737938221
2008	48.07605233749367	14.52881723397035	7.9849753445122252	202.9067898400203
2009	48.5206371593976	14.58867429929725	7.963057562421311	202.0266053704372
2010	49.45265161766456	14.77944655038129	7.997848162516394	202.8730969548354
2011	50.06197620776302	14.91436736034123	8.029549403499225	203.3567501305747
2012	50.30574162836951	14.95255883414477	8.01521149102371	202.6509460370972
2013	50.93949682041676	15.07311215126459	8.03280016100764	202.7160152848625
2014	51.36974691564001	15.16707674176724	8.047974963316133	202.5118344893268
2015	51.98427949585411	15.32241143975537	8.09555247240809	203.5071784110738
2016	52.73262225156111	15.48837673056978	8.136177737444818	204.3876340849885
2017	52.96284958352761	15.53078412614173	8.138373303901918	204.5149399312495
2018	53.32544790034249	15.62132166785267	8.161081054737803	205.2335879942802
2019	53.77219590653053	15.70100879378392	8.166829013457258	205.2211532232022
2020	53.52361794987728	15.61233184265812	8.1108692048836	203.7622718119022
2021	53.68707467864218	15.67445112	8.0951626822222	203.7161239542283
2022	53.35833917802065	15.61828998151096	8.050591529364365	201.7700068767931
2023	53.53564001382598	15.6333676888068	8.025146536078687	201.1416865537782

2024	53.7136375399827	15.64856657553458	7.999837052787909	200.5644926372289
2025	53.90194655330365	15.66663859537353	7.975861175731568	200.0601949484012
2026	54.09456673941283	15.68596088583113	7.952048927106031	199.5998423923955
2027	54.280479002909	15.70531481312809	7.928381412680213	199.1688281480027
2028	54.47924984535974	15.72745612400442	7.905841957289006	198.8035139963368
2029	54.68321100723306	15.75153451775611	7.884592198108197	198.5065497327635
2030	54.89807732116361	15.7793123780207	7.865371765520288	198.2963102403652
2031	55.12231785362374	15.81025720889283	7.847612269092152	198.1499817469928
2032	55.34699162253138	15.84210536806182	7.830444433435472	198.0448057340182
2033	55.5894786575095	15.87822577565442	7.815316274089813	198.0076980026464
2034	55.84213562688187	15.91734509369948	7.801882314853475	198.0392692159848
2035	56.10883293369565	15.96181795946529	7.791281679493411	198.154664162785
2036	56.38939167138339	16.01045012699666	7.782405412992811	198.3325695197726
2037	56.67807132085397	16.06185724106466	7.774899598656138	198.5526108922494
2038	56.98613224844383	16.11764629767337	7.769300874616312	198.8286061977061
2039	57.3075380440058	16.17732420272394	7.765713229010703	199.1620656679102
2040	57.64243148597179	16.24146068102896	7.764255607696247	199.5588416909817
2041	57.9936405630449	16.31021284704994	7.76457139741715	200.0049264404732
2042	58.3572694057325	16.38185427788712	7.765910178201837	200.4819193193021
2043	58.74050234544473	16.45816605767362	7.769214160613818	201.0001488674546
2044	59.13738181549027	16.53774574541114	7.774022631515598	201.5588081531271
2045	59.54729776715446	16.62145463539207	7.780825485617581	202.1634186604425
2046	59.97192368901383	16.70888284733069	7.788953629676573	202.8034697610243
2047	60.40954190318772	16.79963384218616	7.798389599156149	203.4686773315598
2048	60.8627897327625	16.8940525452238	7.809229033333411	204.1622570880854
2049	61.3290178219364	16.99209364565774	7.821635745485956	204.885681823898
2050	61.80680573085126	17.09352115561381	7.835336278130662	205.6395110430576

Table S6: Attributable Risk Factors for ASMR of LOCC in Global and Five SDI Regions in 2021

location_name	val	upper	lower
Global	3.24922491476404	3.92907473387146	2.57853822762883
Global	1.97940409377159	2.60013468302619	1.34949207471181
Global	1.53264141618471	1.93923664358816	1.12449142930289
Global	4.16151119102786	4.84318044095618	3.53492573989261
Global	1.50034344228076	1.92528719753484	1.10303363541414
High-middle SDI	1.94632561518671	2.48182132993654	1.38889914883916
High-middle SDI	1.85407807873931	2.39400585804468	1.29301432735578
High-middle SDI	1.56132833121554	1.95182845090849	1.17723693109792
High-middle SDI	2.84240911382812	3.37927425336008	2.34794150053887
High-middle SDI	0.118016374094121	0.180601693633131	0.0729943303352412
High SDI	1.63533047046553	2.16671185761928	1.11798909992946
High SDI	1.54687391134023	2.09256389645185	1.02312837679261
High SDI	1.6799670495722	2.03555129151184	1.30691430500257

High SDI	2.74338503516534	3.19732212194095	2.27587877824982
High SDI	0.123644281317045	0.203060609248766	0.0682695891632922
Low-middle SDI	7.78883215776079	9.53493021488407	6.25145123958943
Low-middle SDI	3.31148710973588	4.57827758622878	2.17059827975676
Low-middle SDI	1.48967159419394	2.13763486681976	0.935896835888775
Low-middle SDI	8.55513116895371	10.2961884588027	6.96612341603785
Low-middle SDI	5.38874394831896	6.95794700996583	3.9904001677314
Low SDI	4.69351499772472	6.01670632264802	3.54856911157443
Low SDI	1.64928192369995	2.37897797905734	1.02097007999412
Low SDI	1.19878763085693	1.73883953601872	0.716977727555987
Low SDI	5.44522089955007	6.84674246172256	4.21016355535107
Low SDI	3.55074757785416	4.75756378728316	2.46612298092936
Middle SDI	3.01353230505608	3.75306972961621	2.32303294133526
Middle SDI	1.84997556324579	2.46400411820255	1.24577086632383
Middle SDI	1.41863466729443	1.85454138223914	1.01202341071711
Middle SDI	3.82950663013218	4.59390275404574	3.12860759803814
Middle SDI	1.30850073801263	1.77368242754794	0.906127015340527

Abbreviations:

DALY, disability-adjusted life year; GBD, Global Burden of Disease Study;EAPC, estimated annual percentage change;SDI,sociodemographic index;ASIR,age-standardized incidence rate; ASPR, age-standardized prevalence rate; ASMR, age-standardized mortality rate;ASDR,age-standardized DALY rate; UI,uncertainty interval;CI, confidence interval; APC,annual percentage change ;AAPC,average annual percentage change.SDI:sociodemographic index.

© 2025 Li M. et al.
